# A Review on Fused Pyrimidine Systems as EGFR Inhibitors and Their Structure–Activity Relationship

**DOI:** 10.3389/fchem.2022.861288

**Published:** 2022-06-13

**Authors:** Tanuja T. Yadav, Gulam Moin Shaikh, Maushmi S. Kumar, Meena Chintamaneni, Mayur YC

**Affiliations:** Department of Pharmaceutical Chemistry, SPP School of Pharmacy & Technology Management, SVKM’s NMIMS- Deemed to be University, Mumbai, India

**Keywords:** EGFR, pyrimidine, antiproliferative, SAR, fused pyrimidine

## Abstract

Epidermal growth factor receptor (EGFR) belongs to the family of tyrosine kinase that is activated when a specific ligand binds to it. The EGFR plays a vital role in the cellular proliferation process, differentiation, and apoptosis. In the case of cancer, EGFR undergoes uncontrolled auto-phosphorylation that results in increased cellular proliferation and decreased apoptosis, causing cancer promotion. From the literature, it shows that pyrimidine is one of the most commonly studied heterocycles for its antiproliferative activity against EGFR inhibition. The authors have collated some interesting results in the heterocycle-fused pyrimidines that have been studied using different cell lines (sensitive and mutational) and in animal models to determine their activity and potency. It is quite clear that the fused systems are highly effective in inhibiting EGFR activity in cancer cells. Therefore, the structure–activity relationship (SAR) comes into play in determining the nature of the heterocycle and the substituents that are responsible for the increased activity and toxicity. Understanding the SAR of heterocycle-fused pyrimidines will help in getting a better overview of the molecules concerning their activity and potency profile as future EGFR inhibitors.

## 1 Introduction

As per the recent WHO report published in 2020, cancer is the leading cause of mortality in the world. In 2018, the most diagnosed cancer was lung cancer followed by breast cancer (World Health Organization, 2020). The epidermal growth factor receptor (EGFR) belongs to the receptor tyrosine kinase (TK) family and plays an important role in the cancer progression as v-ErbB oncogene present in the avian erythroblastosis virus that has proved to be a mutant homolog of the human EGFR. The similarities were found to exist in the transmembrane and the cytoplasmic domain of the EGFR (Downward et al., 1984; Ullrich et al., 1984).

The EGFR family consists of four members of receptors mainly ErbB1, ErbB2, ErbB3, and ErbB4, and they differ in their binding interactions with the ligand (Teramura et al., 2006). There are 28 distinct combinations of these family members of EGFR that are identified till now (Lemmon and Schlessinger, 2010). Irregularities in the signaling pathway lead to malfunctioning of the EGFR, and various studies have revealed the determining cause of cancer and most of the results correlate to the overexpression of EGFR (Raymond et al., 2000). In normal cells, EGFR expression is believed to be between 40,000 and 100,000 receptors per cell, while cancer cells overexpress EGFR to the tune of more than 1,000,000 receptors per cell (Merlino et al., 1985). The EGFR plays an important role in the cellular proliferation, cell differentiation, angiogenesis, and apoptosis. In cancer, it is observed that EGFR is overexpressed and results in increased cellular proliferation with decreased apoptosis, thereby promoting tumor growth (Liu et al., 2018).

Structurally, EGFR consists of an extracellular ligand-binding site with a dimerization arm (exons 1–16), a transmembrane hydrophobic membrane (exon 17), and an intracellular tyrosine kinase and a C-terminal tail (exons 18–28) (Roskoski, 2014). In the extracellular region, the kinase domain and the C-terminal tail are the mutational “hotspots” under which the EGFR undergoes further mutations. The ectodomain alterations result in the loss of inhibitory regulatory domains that are required for dimerization. It is also worth noting that roughly 20% of glioblastomas have EGFRvIII, which results from the loss of exons 2–7. There is no ligand-dependent signaling for EGFRvIII, although there is a low level of constitutive activity for it. Even though these receptors are not downregulated by endocytosis, the modest constitutive activity is sufficient to enhance signaling in cancer cells (Gan et al., 2013).

**FIGURE 1:**
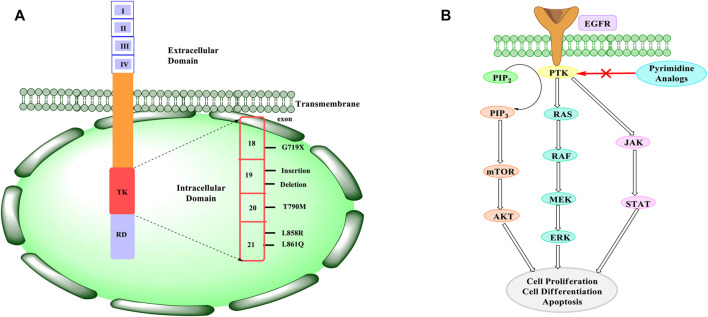
**(A)** EGFR structure and mutations. EGFR consists of three domains namely an extracellular domain, a transmembrane domain, and an intracellular domain. The extracellular domain consists of four subunits that are responsible for binding to the ligand and resulting in dimerization. This results in downstream signaling. The intracellular domain consists of tyrosine kinase (TK) and a regulatory domain (RD), which are responsible for regulating the signaling and physiological process of the EGFR. The TK domain consists of an exon (18–21) that shows mutation. The mutations mainly occur at T790M and L858R. **(B)** Mechanism of inhibiting EGFR. When an EGF ligand binds to the PTK site, it results in the activation of the downstream signalling. Two different pathways are possible. It can activate PI3K pathway or the RAS pathway. PI3K results in the conversion of PIP2 to PIP3, which is further followed by the activation of mTOR and AKT, whereas RAS activation results in the activation of RAF and MEK. Also, there can be activation of JAK-STAT pathway. Whichever pathway it follows, there’s an increase in the cell proliferation, differentiation, and apoptosis of the cell. In cancer, these downstream signaling are enhanced and hence there is uncontrolled cell growth. Therefore, pyrimidine analogues are found to inhibit the PTK site where the EGF ligand binds and results in decreasing the downstream signaling. This reduction in downstream signaling helps in reducing the cell proliferation and differentiation of cancerous cell.

There is roughly 45% of EGFR point exon 19 deletion in the tyrosine kinase domain of the EGFR exon 21 L858R mutation. In the activation loop, the L858R mutation gives a 50-fold greater activity with a higher Michaelis constant (K_m_) compared to the wild-type EGFR (Paez et al., 2004). The EGFR exon 19 in-frame deletions are another type of activating mutation in the kinase domain that is often seen in non-small-cell lung cancer (NSCLC) (Yasuda et al., 2013). In addition to imparting resistance to tyrosine kinase inhibitors, another kinase domain mutant, T790M, is known as the “gatekeeper residue” because of its ability to increase EGFR phosphorylation levels (Yun et al., 2008). The structure of EGFR with possible mutations is shown in Figure 1A.

The EGFR has a PTK (phosphorylated tyrosine kinase) site for attachment of the EGF (epidermal growth factor). For PI3K (phosphoionositide-3-kinase) and RAS, PTK serves as a docking location. When EGFR binds to ligands, it stimulates the PI3K, JAK-STAT, and RAS pathways, which control cell proliferation, transcription, and apoptosis (Liu et al., 2018). The phosphorylation of PIP2 (phosphatidylionositol-4,5-bisphospahte) to PIP3 (phosphatidylionositol-3,4,5-triphospahte) is carried out by PI3K. The AKT is activated because of this conversion, which then activates mTOR that oversees cell proliferation, protein synthesis, cell survival, and transcription, whereas AKT oversees glucose metabolism, cell proliferation, apoptosis, and transcription. When EGFR dimerizes, the RAS pathway is triggered. Also, RAS kinases are activated by dimerization, while RAF kinase is activated by the phosphorylation of MEK (mitogen activated protein kinase). Increased cell proliferation, differentiation, and angiogenesis are caused by the results of both PTK JAK-STAT and RAS activation pathways (Wu and Zhang, 2020). The pathways are elevated in malignant cells, resulting in increased autophosphorylation and cell proliferation, which is followed by reduced apoptosis, promoting cancer progression (Chen et al., 2014). When an inhibitor is given, it binds to EGFR and inhibits downstream signaling, as indicated in Figure 1B. Therefore, inhibiting EGFR will result in reduced cell proliferation, angiogenesis, and metastasis, as well as cell death.

**FIGURE 2:**
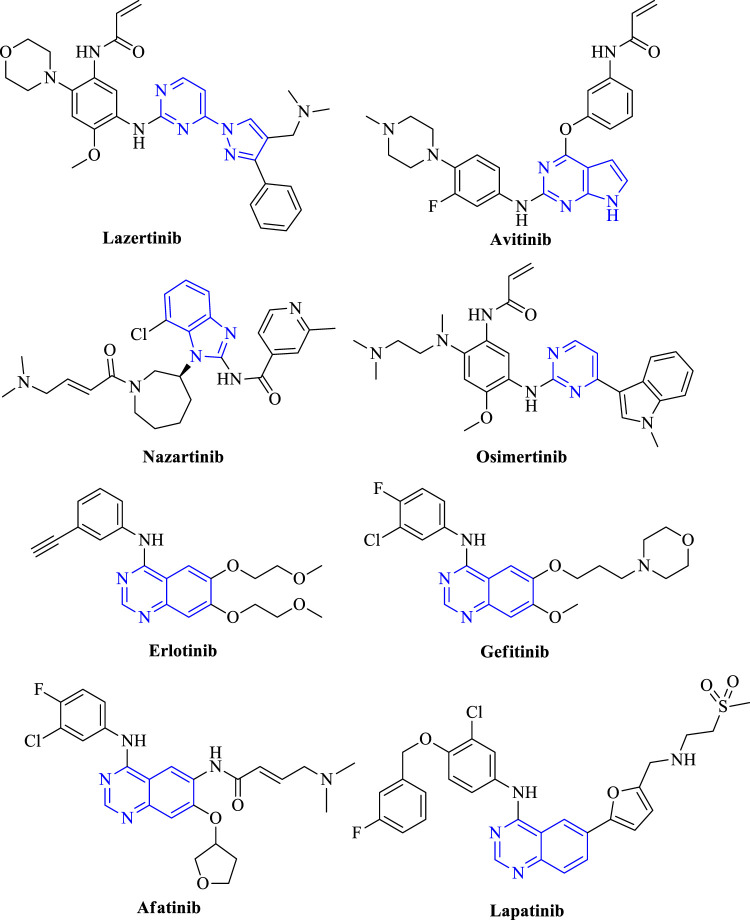
Structures of approved EGFR inhibitors.

The EGFR is widely located in the lung, brain, colon, and breast. It is estimated that 85% of lung cancers include non-small cell lung cancer (NSCLC) and large cell carcinoma (LCC), out of which NSCLC has been deeply understood (Chen et al., 2014). Therefore, inhibiting the EGFR expression can prove beneficial in controlling tumor progression. This results in the development of EGFR kinase inhibitors based on the identified activity from the animal models (Levitzki and Mishani, 2006). The approved list of drugs that are used to target NSCLC includes lazertinib, osimertinib, nazartinib, avitinib, erlotinib, gefitinib, afatinib, and lapatinib. Out of which avitinib is commercially available as a fused-pyrimidine derivative in the treatment of cancer (Figure 2). Lazertinib is a potent irreversible EGFR inhibitor that consists of pyrimidine as a heterocycle (Park et al., 2020; Nagasaka et al., 2021). Osimertinib is a pyrimidine containing molecule that is an oral irreversible inhibitor of wild-type EGFR and mutation T790M. It inhibits EGFR proliferation by binding to the ATP site and hence reduces the downstream signaling of EGFR (Soria et al., 2018; Lazzari et al., 2020). Nazartinib is an indole containing selective EGFR inhibitor that inhibits EGFR^T790M^ and EGFR^L858R^ (Murtuza et al., 2019; Tan et al., 2020). Avitinib is a pyrrolopyrimidine containing mutant-selective T790M EGFR inhibitor (Wang et al., 2020). Erlotinib, gefitinib, afatinib, and lapatinib are all quinazoline-containing EGFR inhibitors. Erlotinib is a reversible inhibitor of EGFR. It binds to ATP binding sites and inhibits EGFR signaling (Abdelgalil et al., 2020). Gefitinib shows selectivity in binding the ATP site of EGFR and inhibiting its autophosphorylation (Giaccone, 2004). Afatinib is an oral irreversible inhibitor of EGFR^WT^ and EGFR^T790M^ that inhibits the kinase domain of EGFR (Wind et al., 2017a; Harvey et al., 2020). Lapatinib is also an oral reversible inhibitor of EGFR and HER2 that inhibits the autophosphorylation and downstream signaling of EGFR (Collins et al., 2019) (refer Table 1).

**TABLE 1:** Details of various EGFR inhibitors and their mechanisms.

Inhibitor	Mechanism of inhibition	References
Lazertinib	Selective toward EGFR mutation	Park et al. (2020)
	Potent irreversible inhibitor	Nagasaka et al. (2021)
Osimertinib	Oral irreversible inhibitor of EGFR^WT^ and EGFR^T790M^	Soria et al. (2018)
	Inhibits ATP binding site of EGFR	Lazzari et al. (2020)
Nazartinib	Mutant-selective irreversible inhibitor of EGFR^T790M^, EGFR^L858R^ exon	Murtuza et al. (2019)
		Tan et al. (2020)
Avitinib	Mutant-selective irreversible inhibitor of EGFR and overcoming T790M advanced NSCLC	Wang et al. (2020)
Erlotinib	Oral, reversible inhibitor of ATP binding site	Abdelgalil et al. (2020)
Gefitinib	Selectively binds to ATP binding site and inhibits autophosphorylation	Giaccone, (2004)
Afatinib	Oral, irreversible inhibitor of EGFR^WT^ and EGFR^T790M^	Wind et al. (2017b)
	Inhibits the kinase domain of EGFR	Harvey et al. (2020)
Lapatinib	Oral, reversible inhibitor of EGFR and HER2	Collins et al. (2019)
	Prevents the autophosphorylation and activation of the signalling pathways	

Gives the details about how the EGFR inhibitors lazertinib, osimertinib, nazartinib, avitinib, erlotinib, gefitinib, afatinib, and lapatinib inhibit the EGFR and their mechanism of action with respect to EGFR mutation.

Various heterocycles have been studied for their anticancer properties, out of which pyrimidine is found to be the most promising one. Most of the approved drugs have pyrimidine heterocycle common in them, hence making pyrimidine an important heterocycle for targeting the inhibition of EGFR. Pyrimidine is a heteroaryl system that consists of nitrogen at the first and third positions. In recent years, pyrimidine has gained focus for its use in many diseases as they have been used as antiviral (Rashad and Ali, 2006; Rashad et al., 2009; Perlíková and Hocek, 2017; Wang et al., 2018), anticancer (Galmarini et al., 2003; Song et al., 2011; Perlíková and Hocek, 2017), anti-hypertensive (El-Hamouly et al., 2006; Ismail et al., 2006), anti-mycobacterial (Garg et al., 2016; Khandazhinskaya et al., 2018), anti-microbial (Okasha et al., 2016; Beyzaei et al., 2017; Marepu et al., 2018), anti-diabetic (Spasov et al., 2017; Fang et al., 2018) and anti-inflammatory activities (Zhang et al., 2017; Abdelgawad et al., 2018)**.**

In the past few years, researchers have synthesized heteroaryl-based pyrimidine derivatives and found that these compounds are active as EGFR inhibitors. One of the recent literatures reports that the pyrimidine system is considered as the bioisosteres to the purine analog of ATP. This results in the better uptake of the pyrimidine compound into the cell and inhibits cell functioning (Abdellatif and Bakr, 2021). Moreover, fused pyrimidines are also reported as CDK inhibitors. The CDK is responsible for the progression of cell cycle. Increase in the CDK activity results in uncontrolled tumor growth (Lee and Han, 2008). As per the reported literatures, pyrimidine fused with different heterocycles such as furan, thiophene, pyrrole, pyrimidine, indole, pyrimidine, acridone, pyrazole, thiazole, and pyridine acts as EGFR inhibitors. Out of which pyrimidine combined with pyrazole (Zhang et al., 2016; Jorda et al., 2019; Massaro et al., 2021), pyrrole (Shi et al., 2021), pyrimidine (El-Moghazy et al., 2011), and pyridine (Barvian et al., 2000; Caballero et al., 2008; Abbas et al., 2019) shows inhibitory activity not only against EGFR but also against CDK. It is suggested that pyrimidine fused with the *N-*containing heterocyclic system acts as a dual inhibitor. However, furopyrimidine, thienopyrimidine, pyrimidoindole, pyrimidoacridone, and thiazolopyrimidine are selective in inhibiting EGFR. This selectivity is due to the presence of a heteroatom O in furan, S in thiophene, N and S in thiazole, N in indole, and an acridone ring system. The focus of this review is to provide an insight into the substituents and structural changes that can be incorporated to find potent EGFR inhibitors.

## 2 Pyrimidines

Pyrimidines have been explored and are widely used heterocycles for determining their activity against various types of cancer, but most reported articles suggest the pyrimidine’s potential in inhibiting EGFR in different types of cancers. This review considers the various fused derivatives of pyrimidine that have been synthesized and are active against EGFR.

## 3 Fused Pyrimidine Derivatives

**FIGURE 3:**
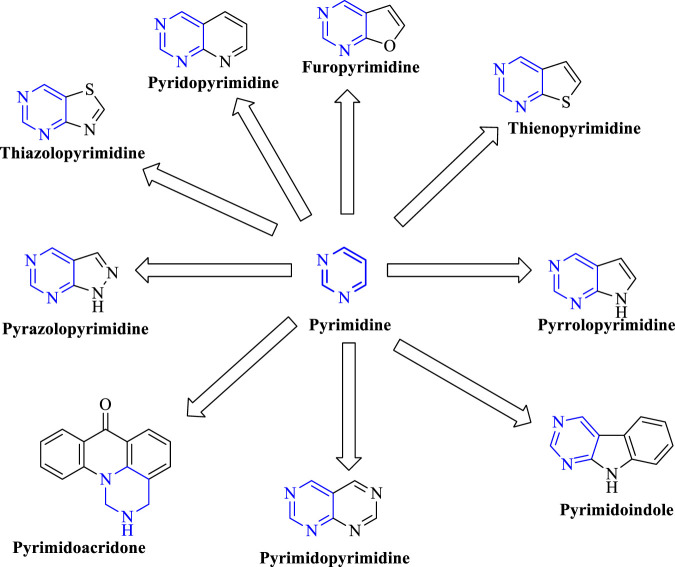
Exhibits various fused-pyrimidine systems that have been reported as EGFR inhibitors..

Pyrimidines can be fused with heterocycles like pyrrole, thiophene, furan, pyran, pyrazole, pyridine, piperidine, acridine, azepine, and even with phenyl rings to obtain a variety of different classes of molecules, which can increase the potency of these heterocycles as anticancer agents. Figure 3 exhibits various fused-pyrimidine systems that have been reported as EGFR inhibitor.

### 3.1 Pyridopyrimidines

Pyridine is a heterocycle that consists of one nitrogen atom in the system. There are a total of five positions where substitution can take place, depending on the fusion of pyridine and pyrimidine.

#### 3.1.1 Disubstituted Pyridopyrimidines

**FIGURE 4:**
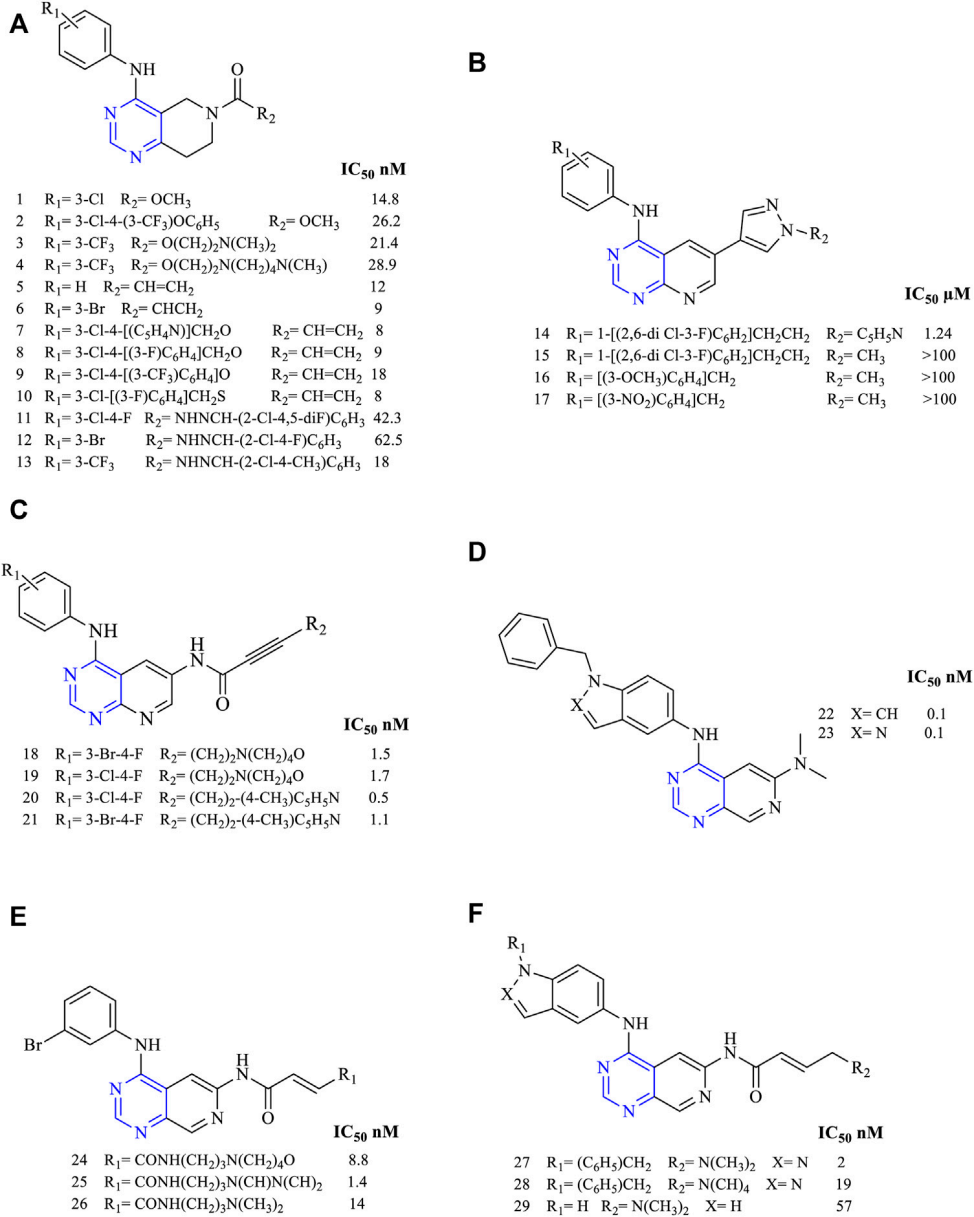
Chemical structure of **(A)** 4-anilino-7,8-dihydropyrido [4,3-*d*]pyrimidine, **(B)** 4,6- disubstituted [2,3-*d*]pyrimidine, **(C)** 4,6- disubstituted [2,3-*d*]pyrimidine, **(D)** 4,6- disubstituted pyrido [3,4-*d*]pyrimidine, **(E)** 4-anilinopyrido [3,4-*d*]pyrimidino-6-acrylamide, and **(F)** 4-fused anilinopyrido [3,4-*d*]pyrimidino-6-acrylamide.

A novel series of 4-anilino-7,8-dihydropyrido [4,3-*d*]pyrimidine (**1–4**, Figure 4A) was designed and synthesized. These molecules were further investigated for their cytotoxicity against A549, HT29, H460, and H1975 and kinase inhibitory activity using EGFR, HER2, and VEGFR. The compounds among the series 1**–**4 were found to be potent against the aforesaid cell lines. Compound **1** showed the highest potency and selectivity to EGFR and HER2 with an IC_50_ of 14.8 and 682 nM, respectively. With the increase in the size of the substituent at the meta position of the phenyl ring, a reduction in the EGFR inhibitory activity was observed (A549 IC_50_ values are 5.67 ± 0.08 µM for **1**, 10.31 ± 0.12 µM for **2,** 9.76 ± 0.08 µM for **3,** and 7.22 ± 0.08 µM for **4**). EGFR IC_50_ values are 14.8 nM for **1**, 26.2 nM for **2**, 21.4 nM for **3,** and 28.9 nM for **4** (Zhang D. et al., 2018). This study indicates that increasing the length of the substituents and such modifications at R_2_ positions can lead to a decrease in the potency of the derivatives.

A study regarding tetrahydropyrido [4,3-*d*]pyrimidine (**5–10**, Figure 4A) for its anticancer activity was investigated against cell lines HT29, A549, H460, and H1975. *In vitro* kinase inhibition studies were carried out using EGFR and HER2 (HT29 IC_50_ 42.38 µM for **5**, 24.62 µM for **6**, 13.87 µM for **7**, 11.69 µM for **8**, 7.91 µM for **9**, and 7.48 µM for **10**; EGFR IC_50_ 12 nM for **5**, 9 nM for **6**, 8 nM for **7**, 9 nM for **8**, 18 nM for **9**, and 8 nM for **10**). Among the series compounds, compounds **5–10** were found to be the most potent in EGFR inhibition with an IC_50_ range of 8–18 nM. Also, this study found that compounds **7–10** were found to inhibit HER2, which is more potent, giving rise to the dual inhibition activity (Zhang Y. et al., 2015). These heterocycles have shown good cytotoxicity in this study.

Recently, a series of tetrahydropyrido [4,3-*d*]pyrimidine (**11–13**, Figure 4A) were studied for anticancer properties using cell lines A549, H1975, MKN-45, and SGC (A549 IC_50_ 7.55 ± 0.41 µM for **11**, 1.61 ± 0.21 µM for **12**, and 4.96 ± 0.34 µM for **13**; EGFR IC_50_ 42.3 nM for 11, 62.5 nM for **12**, and 18 nM for **13**). Further, kinase inhibition studies were carried out for its EGFR, VEFGR2, and EGFR^T790M/L858R^ for its inhibitory potency. Compound **5** was highly potent and selective to EGFR with an IC_50_ of 18 nM, but it was less potent toward VEFGR2 and EGFR^T790M/L858R^ because of the presence of the hydrophobic group trifluoromethyl, which resulted in less activity (Jing et al., 2018).

The series of molecules of 4,6-disubstituted [2,3-*d*]pyrimidine (**14–17**, Figure 4B) have been designed and studied for their anticancer activity, and **14** was found to be more effective in inhibiting MCF-7 and MDA-MB-231 human cancer cell lines (IC_50_ 1.24 µM for 14 and that for 15–17, the values are greater than 100 µM). When studied for its EGFR inhibitory activity, compound **14** was capable of inhibiting EGFR by only 17%, showing less potency toward the enzyme. This was due to the lack of hydrogen bonding with the target, as revealed by the docking simulation studies. The docking simulation was carried using the protein with PDB ID: 4WKQ. It has gefitinib as a co-crystallized ligand. During the docking simulation, it was found that N^1^ of gefitinib showed a hydrogen-bonding MET793 residue and this bonding was absent in **14** and hence the EFGR inhibitory activity was found to be reduced. It shows that hydrogen bonding is important for a molecule to show the EGFR inhibitory activity (Hou et al., 2016).

A study was carried out by synthesizing 4,6-disubstituted [2,3-*d*]pyrimidine (**18–21**, Figure 4C) derivatives for investigating their anticancer activity. Compound **20** was found to be more potent against the NIH3T3 and MDA-MB-453 (EGFR IC_50_ of 1.5 nM for **18**, 1.7 nM for **19,** 0.5 nM for **20**, and 1.1 nM for **21**). Its activity was also determined using ErbB1 and ErbB2, which was found to be more potent toward both the receptors. Western blot analysis suggested that **20** was very selective toward EGFR and showed complete inhibition at 6 nM as compared to the positive control canertinib, which gave complete inhibition at 100 nM (Klutchko et al., 2006), clearly showing compound **20** as a future EGFR inhibitor.

A study carried out on 4,6-disubstituted pyrido [3,4-*d*]pyrimidine (**22–23**, Figure 4D) reported that the pyridopyrimidines were highly potent in inhibiting EGFR and c-ErbB-2, proving them as dual inhibitors (EGFR IC_50_ 0.0001 µM for **22** and **23**). Performing an *ex vivo* study on the BT474 breast tumor xenograft model revealed that the compounds were capable of inhibiting the tumor completely at a dose of 10 mg/kg BID administered for 20 days (Cockerill et al., 2001).

The studies on the class of compounds belonging to 4-anilinopyrido [3,4-*d*]pyrimidino-6-acrylamide (**24–26**, Figure 4E) were found to be highly active against EGFR and ErbB2 (EGFR IC_50_ 8.8 nM for **24**, 14 nM for **25**, and 14 nM for **26**), and hence these classes of compounds were found to be highly potent toward both the kinases. Their potencies could be attributed due to the presence of an acrylamide chain that was capable of interacting with the kinases (Smaill et al., 2001).

A series of 4-fused anilinopyrido [3,4-*d*]pyrimidino-6-acrylamide (**27–29**, Figure 4F) were synthesized (EGFR IC_50_ 2 nM for **27**, 19 nM for **28**, and 57 nM for **29**). Its antiproliferative activity was determined using kinases ErbB1, ErbB2, and ErbB3. Compound **27** was the most potent with activity ranging from 2 to 87 nM. Its pharmacokinetic profile was investigated using rats and monkeys, and it was found to be effective with 60% and 4% bioavailabilities and no mortality of animals observed (Smaill et al., 2016).

##### 3.1.1.1 SAR of Disubstituted Pyridopyrimidine

Depending on the types of substituent and the nature of fusion, disubstituted pyridopyrimidines exhibited a wide range of activity. The following section gives the compilation of SAR:

1) On changing the fused ring from [2,3-*d*] to [3,4-*d*], it showed less inhibitory activity toward EGFR but increased activity against ErbB2 (Smaill et al., 2001) and changing to [4,3-d], which revealed that the molecules obtained were highly specific in EGFR inhibition (Zhang D. et al., 2018).
2) The presence of substituted phenyl amine is required to show inhibitory activity.
3) Substitution at R_1_: the presence of electron-withdrawing substituents like OCH_3_ and NO_2_ exhibited poor inhibition activity (Smaill et al., 2001). Halo substitutions at both (third and fourth) positions showed 2.5–6-fold more potent inhibitory activity toward EGFR than the existing drugs (canertinib, gefitinib, and erlotinib) (Klutchko et al., 2006), whereas mono-substitution with 3-Br gave molecules that showed dual inhibitory activity against EGFR and ErbB2 (Smaill et al., 2001). Increasing the distance between phenyl and nitrogen resulted in less active compounds (Hou et al., 2016). Changing the phenyl ring to indole or indazole gave compounds that were less effective as EGFR inhibitors (Smaill et al., 2016). The presence of benzyloxy and phenoxy increases the selectivity to EGFR rather than to HER2 (Zhang Y. et al., 2015).
4) Substitution at R_2_: the presence of nitrogen (N) is essential for its activity, whereas replacing it with carbon (C) showed a significant loss in its activity (Hou et al., 2016). The presence of tertiary N makes the molecules selective to EGFR (Cockerill et al., 2001), whereas substituting it with C=O retains activity (Zhang D. et al., 2018). The addition of acrylamide (Smaill et al., 2001), benzyloxy (Zhang Y. et al., 2015), phenoxy (Zhang D. et al., 2018), piperidine (Klutchko et al., 2006), and alkene enabled the molecules to exhibit selective inhibition against EGFR, whereas substituting with morpholine and imidazole imbibes a dual inhibitory activity (Smaill et al., 2001).

**FIGURE 5:**
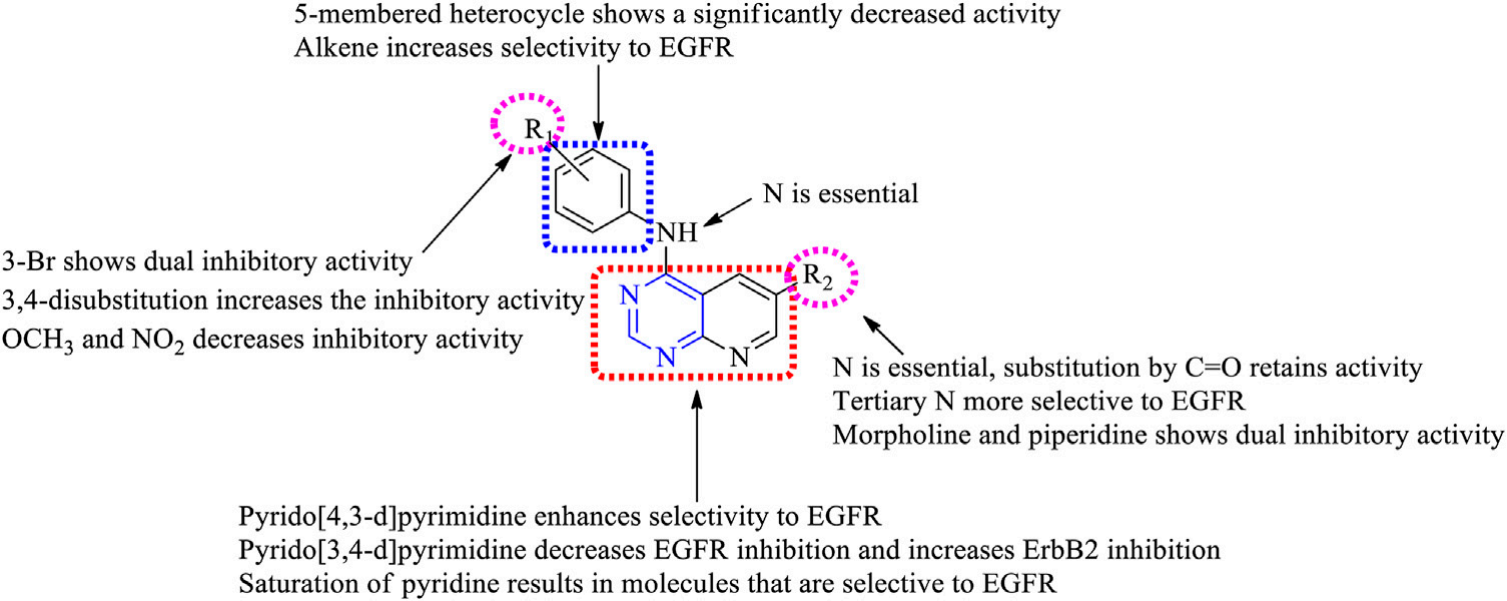
Summary SAR of disubstituted pyridopyrimidine.

The summary of the SAR is depicted in Figure 5.

#### 3.1.2 Trisubstituted Pyridopyrimidines

**FIGURE 6:**
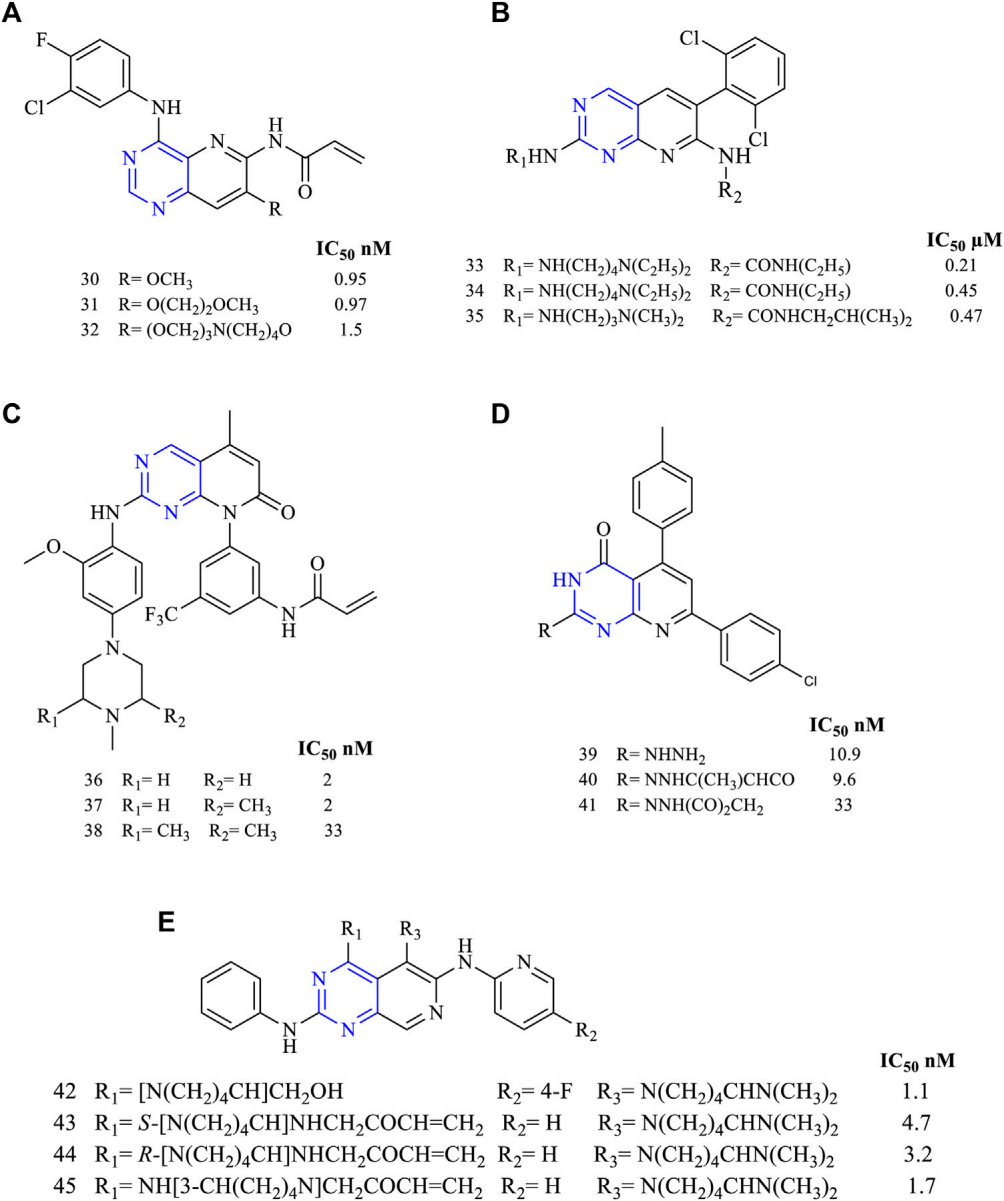
Chemical structure of **(A)** 4,6,7-trisubstituted pyrido [3,2-*d*]pyrimidine-based derivatives, **(B)** 6-aminopyrido [2,3-*d*]pyrimidino-7-urea, **(C)** 2,5,8-trisubstituted pyrido [2,3-*d*]pyrimidine, **(D)** trisubstituted pyrido [2,3-*d*]pyrimidinone, and **(E)** 2,4,6-trisubstituted pyrido [3,4-*d*]pyrimidine.

Studies were conducted by synthesizing 4,6,7-trisubstituted pyrido [3,2-*d*]pyrimidine-based derivatives (**30–32,** Figure 6A) (EGFR IC_50_ 0.95 nM for **30**, 0.97 nM for **31**, and 1.5 nM for **32**). These derivatives’ kinase inhibition activity was determined using A431 cell lines and was found to be irreversible inhibitors of EGFR by using immune-affinity chromatography. A permeability study using the Caco-2 cell line suggested that these molecules have good absorption capacity when compared to vinblastine, but on performing a xenograft study using the nude mice model, it was found to be less effective (Smaill et al., 2000).

A series of 6-aminopyrido [2,3-*d*]pyrimidino-7-urea (**33–35**, Figure 6B) were synthesized to investigate its ability to inhibit receptor TK (EGFR IC_50_ 0.21 µM for **33**, 0.45 µM for **34**, and 0.47 µM for **35**). The studies indicated that these molecules **33–35** were used for studying their inhibitory activities. It was also reported that molecule **33** was highly potent against the MEK/ERK pathway. Also, **33** was effective in suppressing tumors in Colo 205 (Schroeder et al., 2001).

Studies found that 2,5,8-trisubstituted pyrido [2,3-*d*]pyrimidine (**36–38**, Figure 6C) were highly potent in inhibiting EGFR kinase activities with an IC_50_ of 2 nM for **34**, 2 nM for **37**, and 33 nM for **38**. Compound **36** was further studied for its pharmacokinetic properties using the rat model and found to be orally active with a dose of 25 mg/kg. To predict its selectivity, the Western blot experiment revealed that compound **36** was found to be highly selective toward the cell line H1975 (Yu et al., 2017).

A novel series of trisubstituted pyrido [2,3-*d*]pyrimidinone (**39–41**, Figure 6D) were designed and studied for its anticancer activity using different cancer cell lines, and compound **40** was found to be highly potent against HepG-2, PC-3, HCT116, MCF-7, and A549 cell lines at a dose of 100 µM. An IC_50_ value of **40** was found to be around 9.6 µM in A549 cancer cell lines, and further, it was used to study the kinase inhibition activity. The study revealed that **40** was capable of inhibiting 81%–86% of the EGFR activity at a dose of 50 and 100 µM, respectively, suggesting that **40** was showing anticancer activity by strongly inhibiting kinases (Elzahabi et al., 2018).

A new series of 2,4,6-trisubstituted pyrido [3,4-*d*]pyrimidine **(42,** Figure 6E **)** were synthesized and investigated for its antiproliferative activity using HCC827, H1975, and A549 and kinase inhibitory activity using EGFR^L858R^, EGFR^L858R/T790M^, and EGFR^L858R/T790M/C797S^. Compound **42** was found to be more potent in inhibiting EGFR^L858R^, EGFR^L858R/T790M^, and EGFR^L858R/T790M/C797S^, and the IC_50_ values were found to be 1.1, 34, and 7.2 nM, respectively. Docking studies revealed that the hydroxyl group of **42** was interacting with Ser797, representing a stronger interaction with EGFR (Zhang et al., 2018b).

Zhang et al. carried out the studies in determining the specificity in inhibiting EGFR. They designed and synthesized 2,4,6-trisubstituted pyrido [3,4-*d*]pyrimidine (**43–**45, Figure 6E) and investigated for its antiproliferative activity against HCC827, H1975, and A549 cell lines and kinase inhibitory activity using EGFR^L858R^, EGFR^L858R/T790M^, and EGFR^L858R/T790M/C797S^. Compound 45 was found to be more potent against EGFR^L858R^ and EGFR^L858R/T790M^ with an IC_50_ of 1.7 and 23.3 nM, respectively, whereas for EGFR^L858R/T790M/C797S^, IC_50_ was 582.2 nM. The potency of **45** is due to the presence of the acrylamide group and the S configuration of the substituent. Also, an *in vivo* study using a mice model study revealed 45 was the most potent molecule to reduce the tumor growth and reported no mortality (Zhang et al., 2018c).

##### 3.1.2.1 SAR of Trisubstituted Pyridopyrimidine

Depending on the types of substituent and the nature of fusion, trisubstituted pyridopyrimidine exhibits a wide range of activities. Following is the compilation that gives the SAR insights:

1) Maximum inhibitory activity is seen with 2,4,6-trisubstituted [3,4-*d*], 2,5,8-trisubstituted [2,3-*d*] and 4,6,7-trisubstituted [3,2-*d*]. Changing the rings to 2,5,7-trisubstituted [2,3-*d*] showed a decrease in activity.
2) At R_1_: N is essential for its activity. Molecules with 4-(piperazinyl)-phenyl were highly active against the triple mutant EGFR (Yu et al., 2017), whereas substituting with alkyl amino decreases the activity (Schroeder et al., 2001). The presence of pyrazole (Elzahabi et al., 2018) and phenyl resulted in the loss of inhibitory activity (Zhang et al., 2018b).
3) At R_2_: the presence of disubstituted phenyl is highly selective and is an irreversible inhibitor of EGFR (Smaill et al., 2000). The presence of piperidine and pyrrolidine makes the molecule active against the mutant EGFR and shows a dual inhibitory activity (Zhang et al., 2018c).
4) At R_3_: the presence of CH_3_ is active against the mutant (Yu et al., 2017). Substituting with acrylamide improves activity toward EGFR inhibition and also exhibits weak inhibitory activity toward ErbB2 (Smaill et al., 2000). Substituting with the phenyl group results in a significant loss of inhibitory activity (Elzahabi et al., 2018).
5) At R_4_: pyridine is highly active against the mutant form (Zhang et al., 2018b; 2018c) but disubstituted phenyl showed decreased activity (Schroeder et al., 2001).
6) At R_5_: saturating N and substituting with disubstituted phenyl improves activity against the mutant form (Zhang et al., 2018b). The N can be replaced by O, which showed enhanced potency. The presence of the alkoxy group revealed the irreversible inhibition of EGFR and weak activity to ErbB2 (Smaill et al., 2000). Acrylamide showed a significant loss of activity (Schroeder et al., 2001).

**FIGURE 7:**
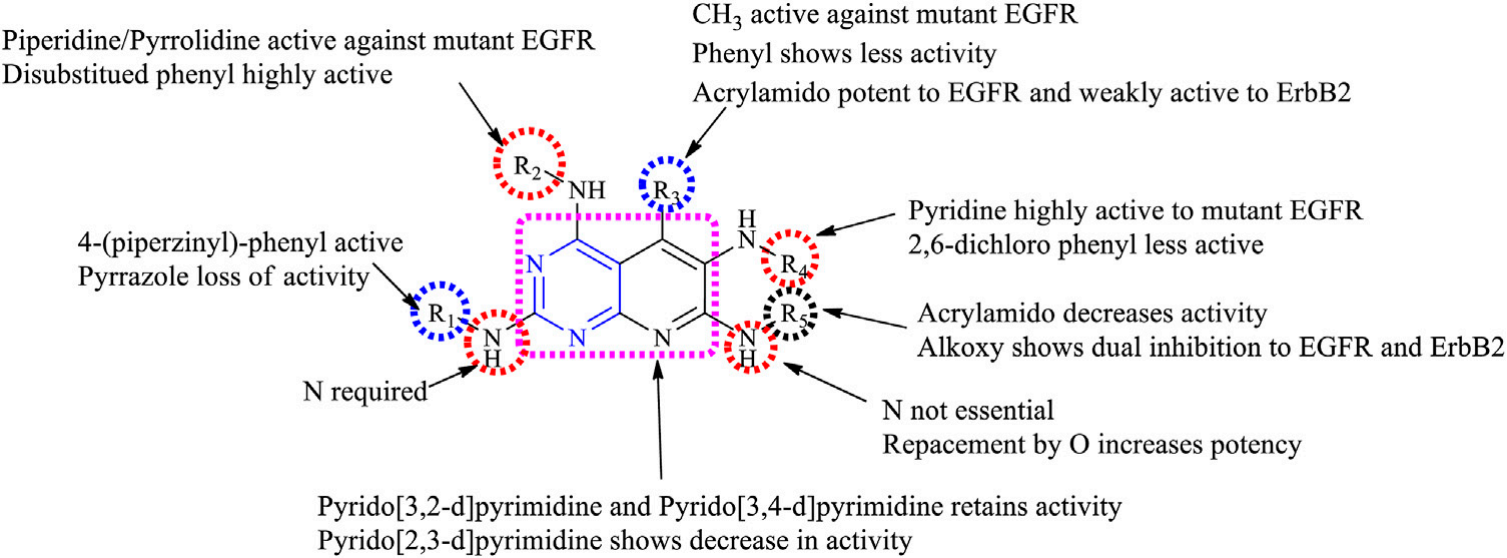
Summary SAR of trisubstituted pyridopyrimidine.

The summary of the SAR is depicted in Figure 7.

### 3.2 Pyrrolopyrimidine

Pyrrole is a five-membered heterocycle that consists of a nitrogen atom in the system. There are a total of five places where substitution can take place, depending on the fusion of pyrrole and pyrimidine rings with different fusion points.

#### 3.2.1 Disubstituted Pyrrolopyrimidine

**FIGURE 8:**
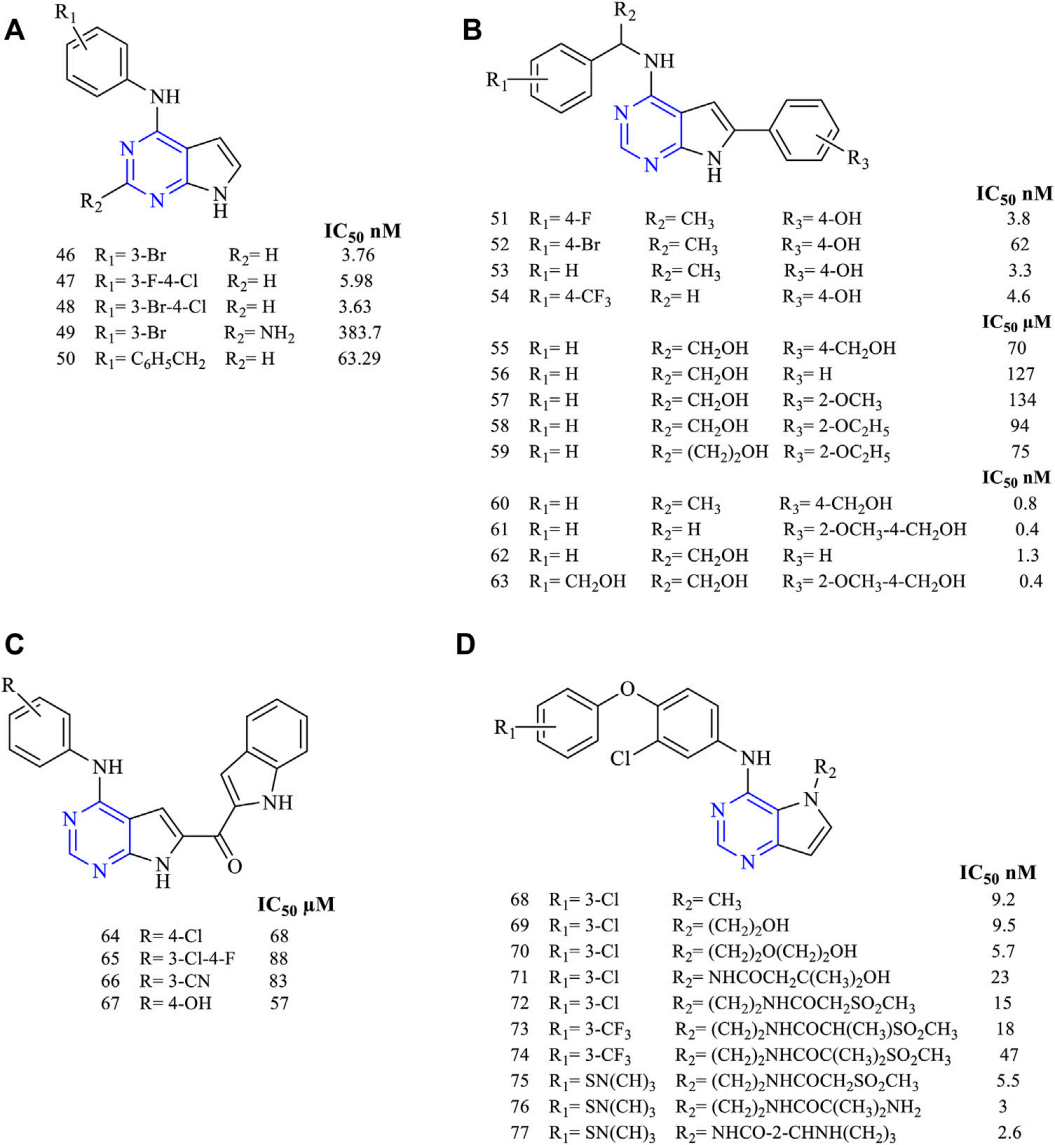
Chemical structure of **(A)** 2,4-disubstituted pyrrolo[2,3-*d*]pyrimidine, **(B)** 4,6-disubstituted pyrrolo[2,3-*d*]pyrimidine, **(C)** 4,6-disubstituted-7H-pyrrolo[2,3-*d*]pyrimidine, and **(D)** 4,5-disubstituted pyrrolo[3,2-*d*]pyrimidine.

Disubstituted pyrrolo [2,3-*d*]pyrimidines have been widely studied for determining their activity against EGFR. A study revealed using 2,4-disubstituted pyrrolo [2,3-*d*]pyrimidine (**46–50**, Figure 8A) proved that the molecules were highly active against EGFR in nanomolar ranges (EGFR IC_50_ 3.76 nM for **46**, 5.98 nM for **47**, 3.63 nM for **48**, 383.7 nM for **49**, and 63.29 nM for **50**). The presence of halogen makes **46** a highly potent EGFR inhibitor with an IC_50_ of 3.76 nM. These molecules also exhibited cytotoxicity toward AURKA cell lines, enabling dual inhibitory activity (Kurup et al., 2018).

The various studies carried out for investigating the EGFR inhibitory activity of 4,6-disubstituted pyrrolo [2,3-*d*]pyrimidines (Figure 8B) showed that the presence of N^4^-phenyl substitutions is necessary for the inhibitory activity. Compounds **51–54** (EGFR IC_50_ 3.8 nM for **51**, 62 nM for **52**, 3.3 nM for **53**, and 4.608 µM for **54**) were found to be highly potent toward Hela cell lines that have shown IC_50_ in the range of 3.3–62 nM (Kaspersen et al., 2011)**.** Substitution on the 6-phenyl in **55–59** (Sundby et al., 2015; Han et al., 2016a) (EGFR IC_50_ 0.07 nM for **55**, 127 µM for **56**, 134 µM for **57**, 94 µM for **58**, and 75 µM for **59**) revealed that they were active against a wide range of cell lines but showed a slight reduction in the activity with an IC_50_ range of 94–127 µM compared to erlotinib (IC_50_ 87 µM) against the EGFR-WT (Han et al., 2016a). Another study showed that the presence of methoxy groups (**60–63**, Figure 8B) (EGFR IC_50_ 0.8 nM for **60**, 0.4 nM for **61**, 1.3 nM for **62** and 0.4 nM for **63**) increased the inhibitory potential of the molecules. Also, they were more potent against the mutant EGFR when compared to the standard erlotinib (Kaspersen et al., 2014).

Becker et al. synthesized 4,6-disubstituted**-**7H-pyrrolo [2,3-*d*]pyrimidines as methanone derivatives (**64–67**, Figure 8C) with a novel approach of attaching indole on the sixth position (EGFR IC_50_ 68 µM for **64**, 88 µM for **65**, 83 µM for **66**, and 57 µM for **67**). They found that these molecules were highly active against EGFR and ErbB2, thereby showing a dual inhibitory activity. Western blot revealed that they were capable of inhibiting the receptors irreversibly (Beckers et al., 2012).

Various studies have concluded that by having substitutions in the fourth and fifth positions, they showed enhanced inhibitory activity. It was also reported that 4,5-disubstituted pyrrolo [3,2-*d*] pyrimidine showed potent antiproliferative activity. Compounds **68–71** (Figure 8D) (EGFR IC_50_ 9.2 nM for **68**, 9.5 nM for **69**, 5.7 nM for **70**, and 23 nM for **71**) proved that they were highly effective in their *in vitro* studies against EGFR and ErbB2 with an IC_50_ range of 5.7–9.5 nM and 2.1–4.1 nM, respectively, thereby exhibiting dual inhibitory activity against EGFR and ErbB2 (Ishikawa et al., 2011).

Compounds **72–74** (Figure 8D) (EGFR IC_50_ 15 nM for **72**, 18 nM for **73,** and 47 nM for **74**) were found to be active against EGFR and ErbB2, but their affinity and selectivity were found to be less in the free state. On converting them into the tosylate salt, these compounds showed improved activity in the rat model (Kawakita et al., 2012b). Compound **71** was further taken up for its preclinical studies and is currently in phase-II clinical trial.

Compounds **75–77** (Figure 8D) (EGFR IC_50_ 5.5 nM for **75**, 3 nM for **76,** and 2.6 nM for **77**) have been reported with high potency and selectivity toward EGFR and ErbB2 with an IC_50_ in the range of 2.6–5.5 nM and 0.92–2 nM, respectively, showing greater activity toward ErbB2. Compound **76** was taken up for further preclinical studies using the xenograft mice model and was reported to be effective at a dose of 50 and 100 mg/kg with no mortality (Kawakita et al., 2012a).

##### 3.2.1.1. SAR of Disubstituted Pyrrolopyrimidine

Depending on the types of substituent and the nature of fusion, disubstituted pyrrolopyrimidine-based molecules exhibit a wide range of activity. The following points are the outcomes, and the SAR for the same are compiled here:

1) Changing the ring from [2,3-*d*] to [3,2-*d*] exhibited dual inhibitory activity toward EGFR and ErbB2 (Ishikawa et al., 2011).
2) At R_1_: the presence of any group decreased its activity. Substitution with NH_2_ decreased its inhibitory activity (Kurup et al., 2018).
3) At R_2_: the presence of N is essential for activity. Increasing the distance between the phenyl and N decreased the activity (Kaspersen et al., 2011). The presence of 4-Br and 4-OH showed good inhibitory activity (Kurup et al., 2018). Substituting with phenyl and 4-CF_3_ resulted in a significant loss of activity (Kaspersen et al., 2014; Sundby et al., 2015; Han et al., 2016a) but substituting it with 4-Cl and 4-CN improved its potency and made the compound active toward EGFR and ErbB2 (Beckers et al., 2012). Substituting with 3-CF_3_ proved the compound to be selective to ErbB2 (Kawakita et al., 2012a).
4) At R_3_: compounds with alkoxy substituents were active (Kawakita et al., 2012b), but the addition of sulfonyl group or substituted side chains significantly reduced the activity (Kawakita et al., 2012a). Pyrrolidine and NH_2_ are well tolerated and showed enhanced activity (Kawakita et al., 2012a).

**FIGURE 9:**
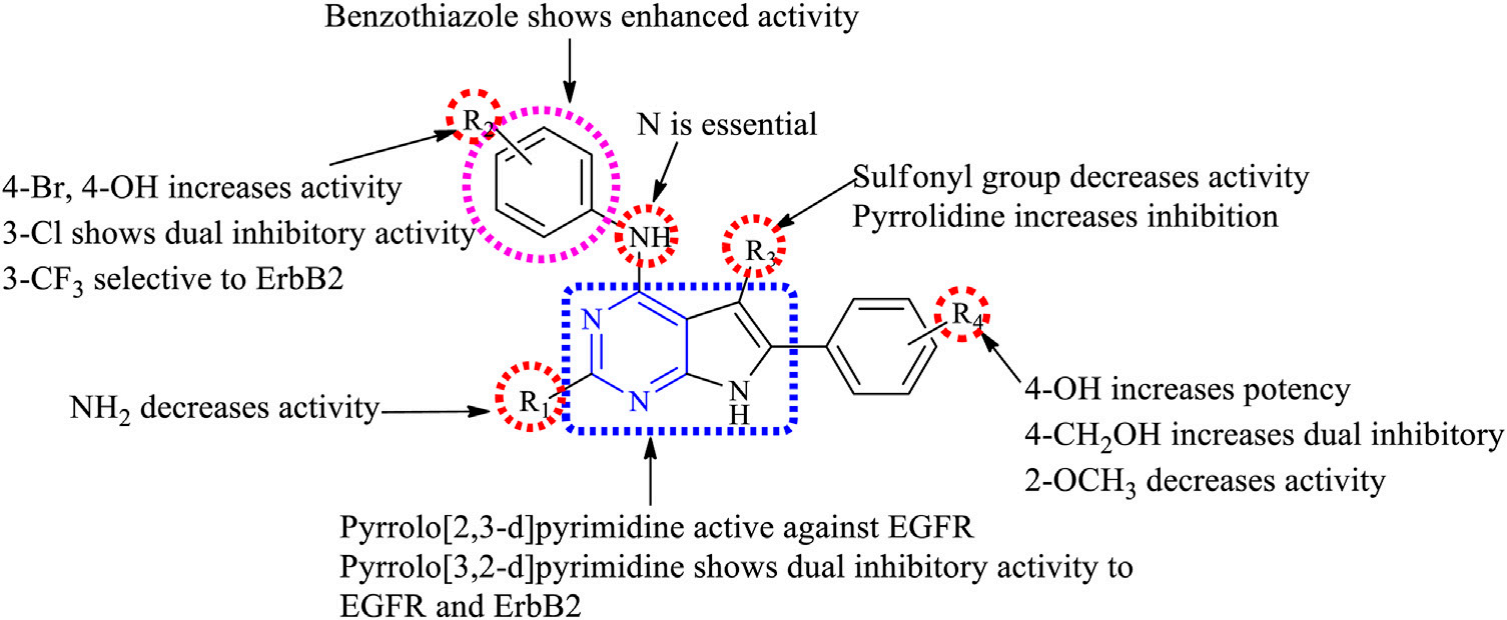
Summary SAR of disubstituted pyrrolopyrimidine.

The summary of the SAR is depicted in Figure 9.

#### 3.2.2 Trisubstituted Pyrrolopyrimidine

**FIGURE 10:**
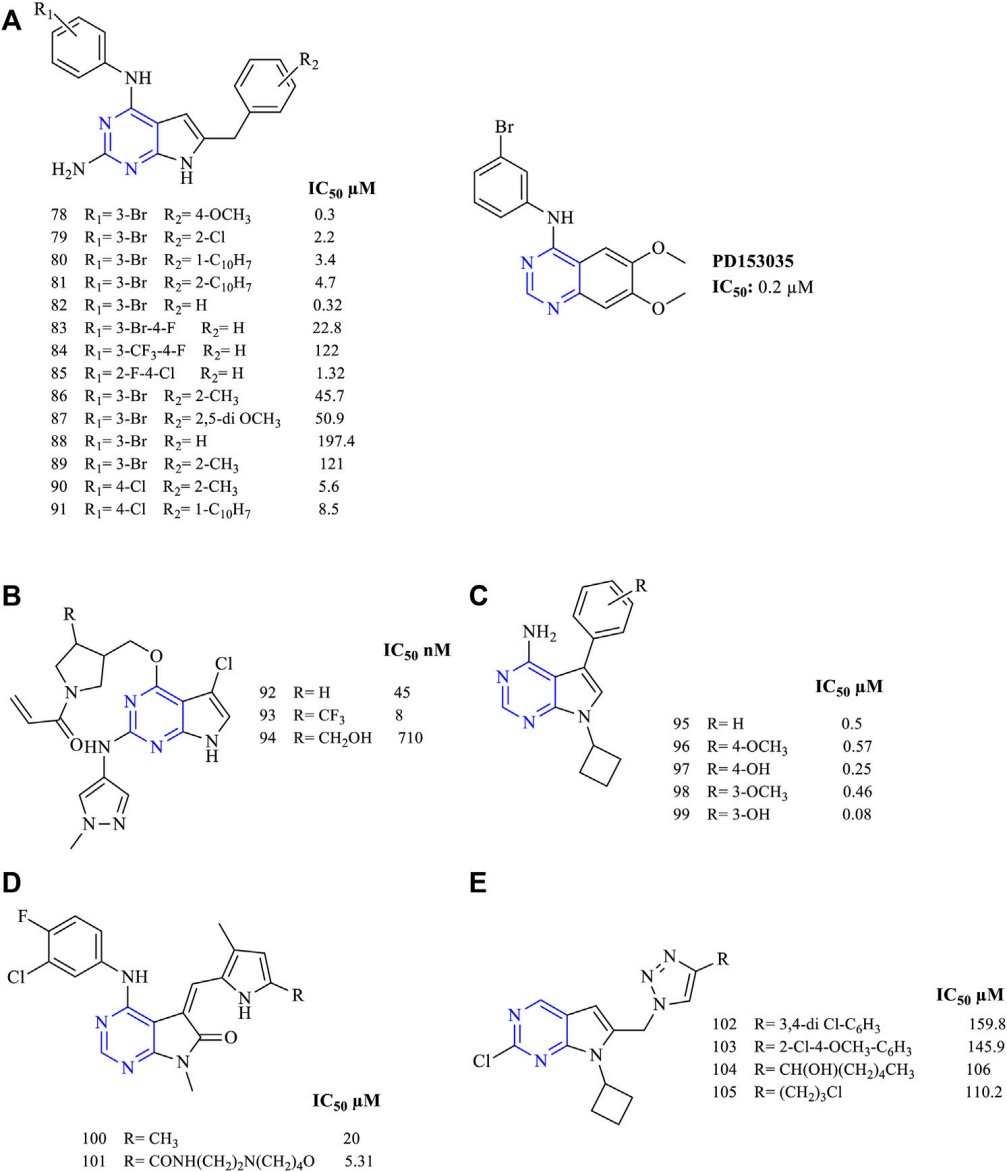
Chemical structure of **(A)** 2,4,6-trisubstituted pyrrolo[2,3-*d*]pyrimidine, **(B)** (2,4,6-trisubstituted pyrrolo[2,3-*d*] pyrimido)-prop-2-en-1-one, **(C)** 4,5,6-trisubstituted pyrrolo[2,3-*d*]pyrimidine, **(D)** 4,5,7-trisubstituted pyrrolo[2,3-*d*]pyrimidine, and **(E)** 2,5,6-trisubstituted pyrrolo[2,3-*d*]pyrimidine.

A wide range of trisubstituted pyrrolopyrimidine were synthesized and studied for their antiproliferative activities against EGFR. All the trisubstituted pyrrolopyrimidine compounds showed the presence of pyrrolo [2,3-*d*]pyrimidine fused rings. The 2,4,6-trisubstituted pyrrolo [2,3-*d*]pyrimidine derivatives also showed that they were cytotoxic against the cancer cell lines. A study indicating **78–81** (Figure 10A) reported that their activity was comparable to the standard used (PD153035) in the studies (EGFR IC_50_ values are 0.2 µM for PD153035, 0.3 µM for **78**, 2.2 µM for **79**, 3.4 µM for **80,** and 4.7 µM for **81)** (Gangjee et al., 2008). Similar results were also reported using the A431 cell line for the identification of cytotoxic studies. Compounds **82–85** (Figure 10A) (EGFR IC_50_ 0.32 µM for **82**, 22.8 µM for **83**, 122 µM for **84,** and 1.32 µM for **85**) exhibited good cytotoxic activity and EGFR inhibitory activity (Gangjee et al., 2010a). By varying the substitution at the R_2_ position with groups like methoxy and chloro, compounds **86–91** (IC_50_ 45.7 µM for **86**, 50.9 µM for **87**, 197.4 µM for **88**, 121 µM for **89**, 5.6 µM for **90**, and 8.5 µM for **91**) significantly reduced its activity (Figure 10A) (Gangjee et al., 2010b, 2012).

This study reported the design and synthesis of (2,4,6-trisubstituted pyrrolo [2,3-*d*] pyrimido)-prop-2-en-1-one for the antiproliferative action. Compounds **92–94** (Figure 10B) proved that they are highly effective against the mutant EGFR cell line with an IC_50_ of 4.5–710 nM. The presence of 1,2-diazole helped in improving its activity by inhibiting the EGFR (Cheng et al., 2016).

Substitution of hydroxy and methoxy substituents at R_2_ positions on the phenyl ring yielded compounds that exhibited high selectivity in inhibiting EGFR. Compounds **95–99** (Figure 10C) (EGFR IC_50_ 0.5 µM for **95**, 0.57 µM for **96**, 0.25 µM for **97**, 0.46 µM for **98,** and 0.08 µM for **99**) were found to be highly selective to EGFR, which showed that this type of action and this activity may be due to the presence of a cyclobutyl substituent at the seventh position. The cycloalkyl ring present at the N^7^ position resembles the ribose moiety of ATP, and hence the cyclobutyl ring is capable of inhibiting the ATP binding site resulting in higher EGFR inhibitory activity (Widler et al., 2001).

A study of trisubstituted pyrrolo [2,3-*d*]pyrimidine substituted with methyl-pyrrole (**100–101**, Figure 10D) (EGFR IC_50_ 20 µM for **100**, and 5.31 for **101**) showed that the compounds were less active in inhibiting EGFR (Sun et al., 2002).

This study indicated the design and synthesis of 2,5,6-trisubtituted pyrrolo [2,3-*d*]pyrimidine compounds **102–105** (Figure 10E) (EGFR IC_50_ 159.8 µM for **102**, 145.9 µM for **103**, 106 µM for **104,** and 110.2 µM for **105**). The study outcome was that the aryl and the alkyl functionalities can be proved to be beneficial for its anticancer cytotoxicity. Also, the presence of triazole helped in improving its anti-inhibitory activity (Thiriveedhi et al., 2019).

##### 3.2.2.1 SAR of Trisubstituted Pyrrolopyrimidine

Depending on the type of substituents and the nature of fusion, trisubstituted pyrrolopyrimidine compounds exhibited a wide range of activity. The following section gives the compilation of the SAR:

1) Pyrrolo [2,3-*d*]pyrimidine is reported to show anticancer activity.
2) At R_1_: the presence of amino (Gangjee et al., 2008; 2010a; 2010b, 2012) and substituted amino (Cheng et al., 2016) provides good anticancer activity against the cell lines. Substituting with Cl significantly reduces the activity (Thiriveedhi et al., 2019).
3) At R_2_: 3-Br is required for pyrrolopyrimidine to exhibit the EGFR inhibitory activity (Gangjee et al., 2008). Substituting with ethynyl or CF_3_ results in a significant loss of activity (Gangjee et al., 2010a). The 4-Cl substitution is effective against EGFR (Gangjee et al., 2012); however, pyrrolidine makes the compound highly selective and effective (Cheng et al., 2016).
4) At R_3_: substituting phenyl with OH and OCH_3_ makes the compound highly selective and increases the potency (Widler et al., 2001). Substitution with methyl-pyrrole, however, reduces the activity (Sun et al., 2002).
5) At R_4_: the presence of phenyl moiety is responsible for its biological activity (Gangjee et al., 2008). The presence of naphthyl coupled with 3-Br at R_2_ showed significant improvement in the activity (Gangjee et al., 2008; 2010b). However, substituting with triazole retains the activity (Thiriveedhi et al., 2019).
6) At R_5_: the cyclobutyl retains the activity and also helps in improving its pharmacokinetic properties by delaying metabolism (Widler et al., 2001; Thiriveedhi et al., 2019); however, changing to CH_3_ reduces its activity (Sun et al., 2002).

**FIGURE 11:**
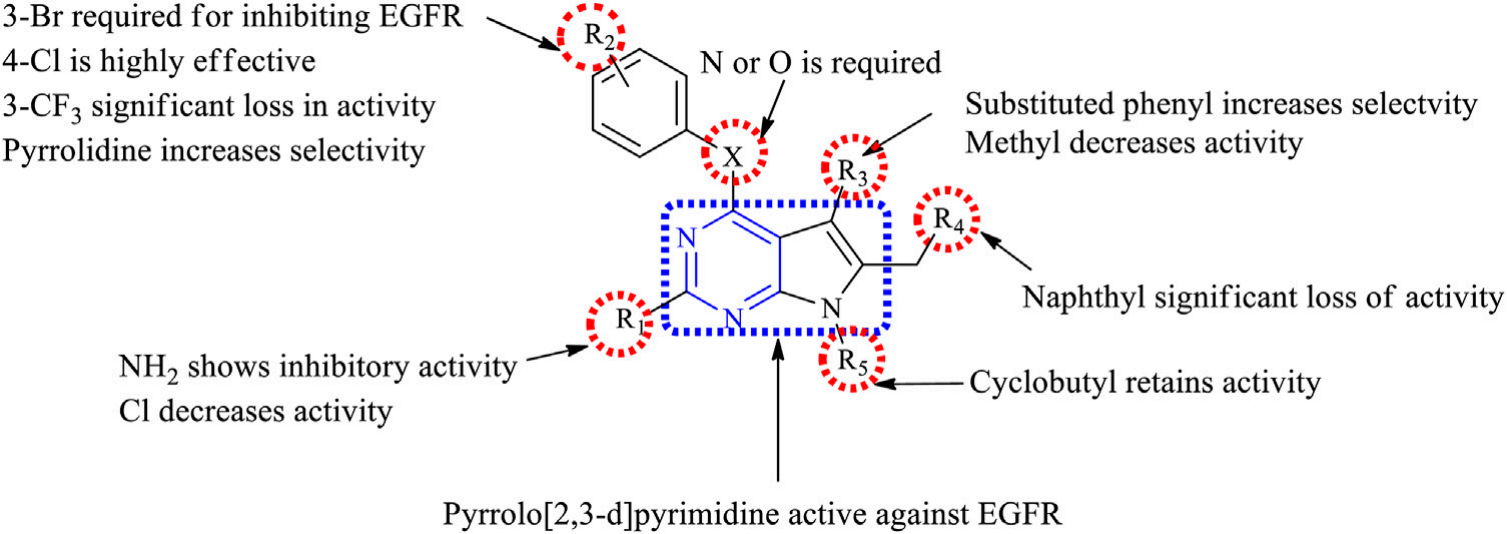
Summary SAR of trisubstituted pyrrolopyrimidine.

The summary of the SAR is depicted in Figure 11.

### 3.3 Thienopyrimidine

Thiophene is a five-membered heterocycle that consists of a sulfur atom in the system. The literature reported that the fourth and sixth positions have been used for substitutions and studies have been conducted for determining their anticancer potency.

#### 3.3.1 Disubstituted Thieno[2,3-*d*]Pyrimidine

**FIGURE 12:**
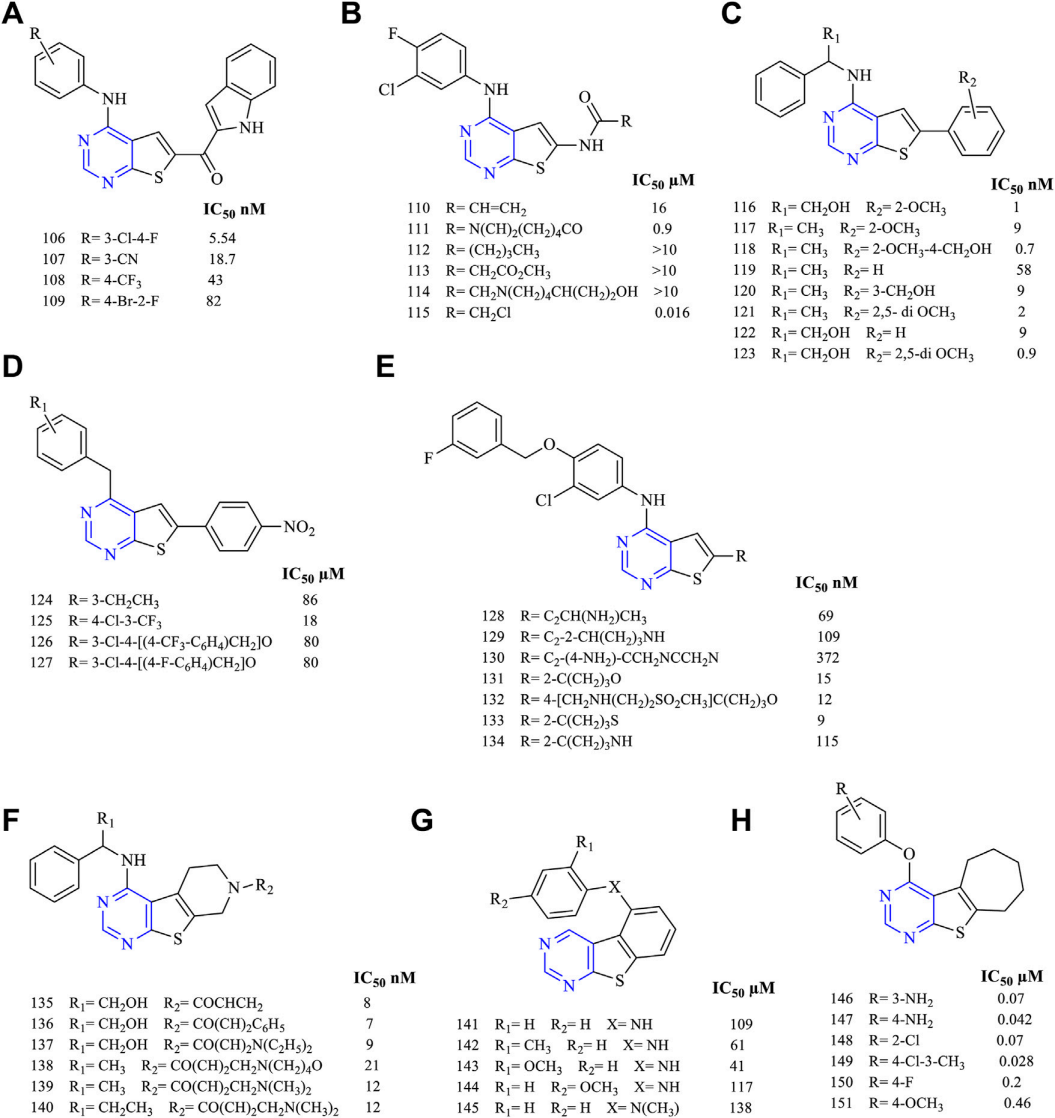
Chemical structure of **(A)** 4-substituted anilinothieno[2,3-*d*]pyrimidine-6-methanone, **(B)** 4-substituted anilinothieno[2,3-*d*]pyrimidin-6-amide, **(C)** 4-substituted amino-6-phenylthieno[2,3-*d*]pyrimidine, **(D)** 4-anilino-6-phenylthieno[2,3-*d*]pyrimidine, **(E)** substituted phenyl thieno[2,3-*d*]pyrimidine, **(F)** fused pyridine thieno[2,3-*d*]pyrimidine, **(G)** fused substituted phenyl thieno[2,3-*d*]pyrimidine, and **(H)** fused cycloheptane thieno[2,3-*d*]pyrimidine.

The compound 4,6-disubstituted thieno [2,3-*d*]pyrimidine has been widely studied for its anticancer activity. The literature suggests that this moiety is beneficial for exhibiting anticancer activity via inhibition of EGFR. A study on 4-substituted anilinothieno [2,3-*d*] pyrimidine-6-methanone (**106–109**, Figure 12A) (EGFR IC_50_ 5.54 nM for **106**, 18.7 nM for **107**, 43 nM for **108,** and 82 nM for **109**)-based derivatives was found to be a dual inhibitor of EGFR and ErbB2. Further studies were carried out using a mutant and wild-type EGFR kinase and the selectivity profile was calculated. It was found that compounds **106** and **108** were found to be highly potent to EGFR and ErbB2, respectively, coupled with good inhibitory activity (Beckers et al., 2012).

A series of 4-substituted anilinothieno [2,3-*d*] pyrimidin-6-amide (**110–115**, Figure 12B) (EGFR IC_50_ 16 µM for **110**, 0.9 µM for **111**, >10 µM for **112–114,** and 0.016 µM for **115**) were synthesized and subjected to inhibitory studies. The IC_50_ was determined using EGFR^WT^ and EGFR^T790M/L858R^. It was reported that **111** was highly potent and effective (IC_50_ 0.9 nM for EGFR^WT^ and IC_50_ 4 nM for EGFR^T790M/L858R^). Also, compounds **110** and **111** were found to be active against ErbB2, suggesting a dual inhibitory activity (Ji et al., 2014).

It was also investigated that 4-substituted amino-6-phenylthieno [2,3-*d*]pyrimidine (**116–123**, Figure 12C) (EGFR IC_50_ 1 nM for **116**, 9 nM for **117**, 0.7 nM for **118**, 58 nM for **119**, 9 nM for **120**, 2 nM for **121**, 9 nM for **122,** and 0.9 nM for **123**) was found to be less effective except compound **118**. The compounds are erlotinib derivatives with an additional aromatic ring that makes the compounds less effective irrespective of the similar dock pose. The potency of **118** (IC_50_ 0.7 nM) was very close to erlotinib (IC_50_ 0.4 nM) but was found to be more cytotoxic (Bugge et al., 2015, 2016).

The studies have further reported that 4-anilino-6-phenylthieno [2,3-*d*]pyrimidine (**124–127**, Figure 12D) (EGFR IC_50_ 86 µM for **124**, 18 µM for **125**, 80 µM for **126,** and 86 µM for **127**) was found to be effective in kinase inhibitory activity and proved compounds were exhibiting dual inhibitory characteristics against EGFR and ErbB2. Compounds **126** and **127** were found to be more cytotoxic than lapatinib (Milik et al., 2018).

To understand the inhibitory activity of thienopyrimidine, compounds with phenyl alkynyl substituent (**128–130**, Figure 12E) (EGFR IC_50_ 69 nm for **128**, 109 nM for **129,** and 372 nM for **130**) and heteroaryl substituent (**131–134**, Figure 12E) (EGFR IC_50_ 15 nM for **131**, 12 nM for **132**, 9 nM for **133,** and 115 nM for **134**) were studied. Both types of substituents were capable of inhibiting EGFR and ErbB2 in the kinase study. The presence of a hetero-aromatic system yielded better inhibition activity, and **112** was found to be more effective and potent (Wood et al., 2008; Rheault et al., 2009).

Further studies suggested that when thienopyrimidine was fused with a ring (saturated ring or an aromatic ring), there was an improvement in its inhibition activity. The fusion of pyrido with thieno [2,3-*d*]pyrimidine resulted in compounds **135–140** (Figure 12F) (EGFR IC_50_ 8 nM for **135**, 7 nM for **136**, 9 nM for **137**, 21 nM for **138**, 12 nM for **139–140,** and 20 nM for gefitinib) that were highly potent against the double mutant EGFR cell line. Moreover, **135** was found to be more selective against EGFR from Western blotting analysis. Compound **136** was further taken up for preclinical studies using the xenograft mice model, which proved that they was a significant decrease in the tumor size (Wu et al., 2010).

It was found that fusing the substituted phenyl with thieno [2,3-*d*]pyrimidine resulted in compounds **141–145** (Figure 12G) (IC_50_ 109 µM for **141**, 61 µM for **142**, 41 µM for **143**, 117 µM for **144,** and 138 µM for **145**) that were highly effective against the glioblastoma. Compound **143** exhibited a strong inhibition against the cell lines U87-MG and DBTRG.05-MG. The results obtained from the Western blot analysis proved its selectivity toward glioblastoma cell line DBTRG.05-MG harboring EGFR, which showed that compound **143** has the capability of inhibiting the EGFR downstream signaling (Pédeboscq et al., 2010).

A recent study proposed that the fusion of cycloheptane with thieno [2,3-*d*]pyrimidine resulted in compounds **146–151** (Figure 12H) (EGFR IC_50_ 0.07 µM for **146**, 0.042 µM for **147**, 0.07 µM for 148, 0.028 µM for **149**, 0.2 µM for **150,** and 0.46 µM for **151**) that were capable of showing promising results in inhibiting EGFR and VEGFR. The IC_50_ of **147** (0.042 µM) obtained was found to be potent compared with erlotinib (0.03 µM), whereas **149** showed the highest apoptotic activity against the VEGFR (Mghwary et al., 2019).

#### 3.3.2 Disubstituted Thieno[3,2-*d*]Pyrimidine

**FIGURE 13:**
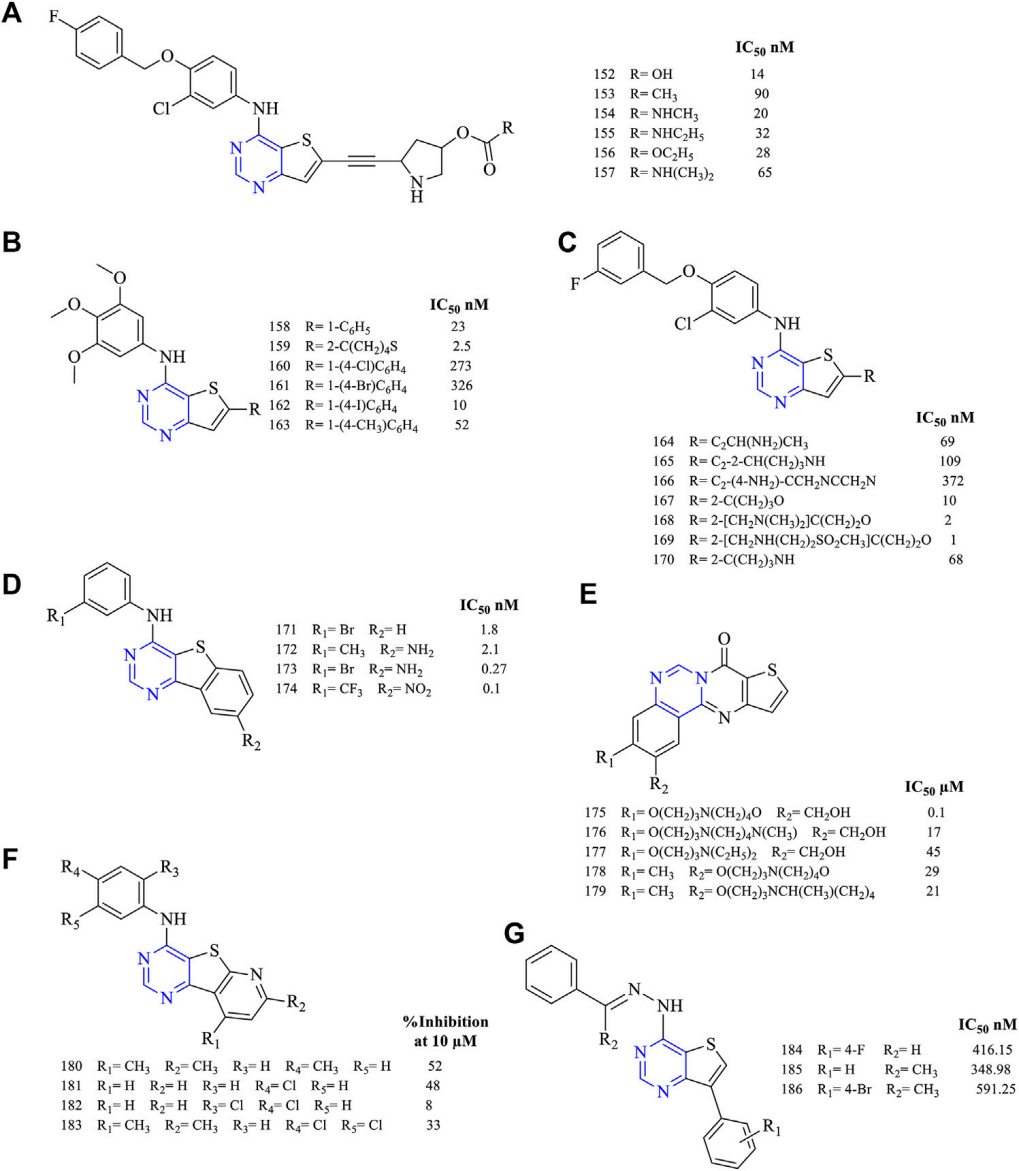
Chemical structure of **(A–C)** 4,6-disubstituted thieno[3,2-*d*]pyrimidines, **(D)** phenyl fused thieno[2,3-*d*]pyrimidines, **(E)** quinazoline fused thieno[2,3-*d*]pyrimidines, and **(F)** pyrido fused thieno[2,3-*d*]pyrimidines. **(G)**: 4,7-disubstitued thieno [3,2-*d*]pyrimidine.

A series of 4,6-disubstituted thieno [3,2-*d*]pyrimidines were synthesized and studied for their anticancer activity. The results proved that the pyrrolidinyl-acetylenic thieno [3,2-*d*]pyrimidine (**152–157**, Figure 13A) (EGFR IC_50_ 14 nM for **152**, 90 nM for **153**, 20 nM for **154**, 32 nM for **155**, 28 nM for **156,** and 65 nM for **157**) was found to have not only good inhibitory properties but also good covalent binding with EGFR. These compounds exhibited a dual inhibitory potential toward EGFR and ErbB2 (Hubbard et al., 2008). It was further reported that modifying the carbamate helps in improving the inhibitory activity and enhances the oral bioavailability of the same (Stevens et al., 2009).

The presence of substituted aniline (**158–163**, Figure 13B) (EGFR IC_50_ 23 nM for **158**, 2.5 nM for **159**, 273 nM for **160**, 326 nM for **161**, 10 nM for **162,** and 52 nM for **163**) was reported to show good inhibitory activity against EGFR but lacked activity toward VEGFR. Moreover, **159** has shown promising results in inhibiting EGFR with slight activity toward tubulin inhibition. Western blot has proved that **163** was a selective inhibitor of VEGFR (Romagnoli et al., 2019).

The alkynyl group helps in improving the inhibitory activity (**164–170**, Figure 13C) (EGFR IC_50_ 69 nM for **164**, 109 nM for **165**, 372 nM for **166**, 10 nM for **167**, 2 nM for **168**, 1 nM for **169,** and 68 nM for **170**). Depending on the type of group attached, the activity varies. The presence of a linker between the aryl and the parent rings helps in bringing selectivity to the compounds. Compound **165** exhibited only 24% alkylation to EGFR, whereas the mesylate group was found to be a potent EGFR inhibitor (Wood et al., 2008; Rheault et al., 2009).

From the literature, it was found that the fused thieno [2,3-*d*]pyrimidines were investigated for their inhibitory potential. The fusion of phenyl rings (**171–174**, Figure 13D) (EGFR IC_50_ 1.8 nM for **171**, 2.1 nM for **172**, 0.27 nM for **173,** and 0.1 nM for **174**) resulted in compounds with an IC_50_ range of 0.27–2.1 nM, suggesting that the compounds were highly effective in inhibiting EGFR. Further, **173** was taken in preclinical studies, which was found to be disappointing due to the ineffective dose (50 mg/kg) and less solubility (less than 30 μg/ml) (Showalter et al., 1999).

Quinazoline-fused thieno [3,2-*d*]pyrimidine (**175–179**, Figure 13E) (DU-145 IC_50_ 0.1 µM for **175**, 17 µM for **176**, 45 µM for **177**, 29 µM for **178,** and 21 µM for **179**) compounds reported EGFR inhibitory activity. Their inhibitory potential was determined using DU-145 and MiaPaCa-2 cell lines but was found to be less effective as they showed the inhibition of a maximum of 10% (Zheng et al., 2010).

Studies on pyrido-fused thieno [3,2-*d*]pyrimidine (**180–183**, Figure 13F) (percent EGFR inhibition at 10 µM; 52% for **180**, 48% for **181**, 8% for **182,** and 33% for **183**) reported showing EGFR inhibitory activity. Compound **181** was very potent as it exhibited an 81% inhibitory potential when compared to doxorubicin. It was further taken up for preclinical studies but failed due to a lack of specificity in the study model (Aziz et al., 2015).

It was found that thieno [3,2-d]pyrimidine (**184–186**, Figure 13G) (EGFR IC_50_ 416.15 nM for **184**, 348.98 nM for **185,** and 591.25 nM for **186**) are EGFR inhibitors. These compound**s** were found to be less potent in inhibiting EGFR when compared to the reference erlotinib (EGFR IC_50_ 166.92 nM) (Traxler et al., 1997).

#### 3.3.3 SAR of Thienopyrimidine

Depending on the type of substituents, thienopyrimidine exhibits EGFR inhibitory activity. It was also reported that thieno [2,3-*d*]pyrimidine showed inhibitory activity against EGFR, whereas thieno [3,2-d]pyrimidine shows a dual inhibitory activity toward EGFR and ErbB-2. The following is the compilation of the SAR:

1) At R_1_: the presence of a nitrogen atom is essential for inhibitory activity. The presence of substituted phenyl improves the activity. The substitution of F and Cl showed optimal activity (Ji et al., 2014). Increasing the distance between the N and phenyl groups helps in improving the selectivity toward EGFR (Bugge et al., 2015, 2016). The substitution with the phenoxy phenyl group shows very potent inhibitory activity (Hubbard et al., 2008; Wood et al., 2008; Rheault et al., 2009; Stevens et al., 2009; Pédeboscq et al., 2010; Wu et al., 2010; Milik et al., 2018; Mghwary et al., 2019). The presence of CH_2_OH increased its selectivity and activity (Wu et al., 2010). Substitutions with CH_3_ and OCH_3_ showed a strong blocking of EGFR (Pédeboscq et al., 2010; Romagnoli et al., 2019), whereas substitution by the NH_2_ group showed very potent activity and strong inhibition (Mghwary et al., 2019). The presence of the CH_3_ group helps in EGFR inhibitory activity. The presence of Br on the phenyl ring has shown enhanced potency in inhibiting EGFR as compared to that of F (Traxler et al., 1997).
2) At R_2_: the presence of an indole ring makes the compound show a dual inhibitory activity (Beckers et al., 2012). The presence of an amide linkage makes the molecule more active (Ji et al., 2014). The addition of acrylamide in thieno [2,3-*d*]pyrimidine makes the compound very potent. The substituted aryl showed increased activity and selectivity to EGFR (Bugge et al., 2015, 2016; Milik et al., 2018), whereas in the case of thieno [3,2-*d*]pyrimidine, it showed the loss of activity (Romagnoli et al., 2019). The methoxy group exhibits inhibitory potential (Bugge et al., 2016), whereas the nitro group decreases the activity due to its strong electron-withdrawing abilities (Milik et al., 2018). The thiophene-substituted derivatives were also effective in inhibiting EGFR and VEGFR (Romagnoli et al., 2019). The alkynyl group of thieno [2,3-*d*]pyrimidine was dually effective in inhibiting EGFR and ErbB2 (Wood et al., 2008; Rheault et al., 2009), but in the case of thieno [3,2-*d*]pyrimidine, the selectivity for ErbB2 decreased significantly (Wood et al., 2008; Rheault et al., 2009). The smaller heteroaryl-like pyrrole showed good dual inhibitory potential (Hubbard et al., 2008), but replacing it with a morpholine group reduced its activity (Stevens et al., 2009).
3) The fusion of thienopyrimidine with other rings helps in improving its activity. The fusion with piperidine (Wu et al., 2010), phenyl (Showalter et al., 1999; Pédeboscq et al., 2010), and cycloheptane (Mghwary et al., 2019) was found to exhibit a dual inhibitory activity. The presence of quinazoline fusion also improved the activity of the compound (Zheng et al., 2010). The pyrido fused molecules were found to be weakly active (Aziz et al., 2015).

**FIGURE 14:**
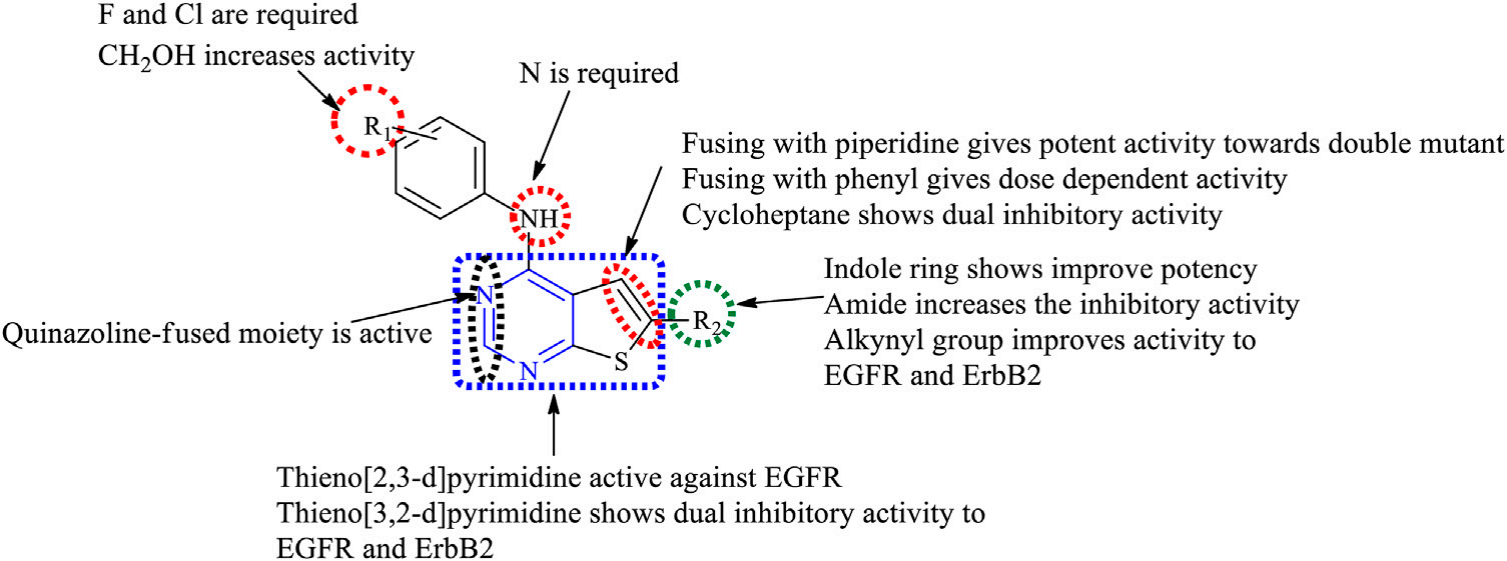
Summary SAR of thienopyrimidine.

The summary of the SAR is depicted in Figure 14.

### 3.4 Pyrazolopyrimidine

Pyrazole is a five-membered heterocycle that consists of two nitrogen atoms in the system. The literature reports that pyrazolo [3,4-d]pyrimidines have been synthesized and their EGFR inhibition studies were carried out.

**FIGURE 15:**
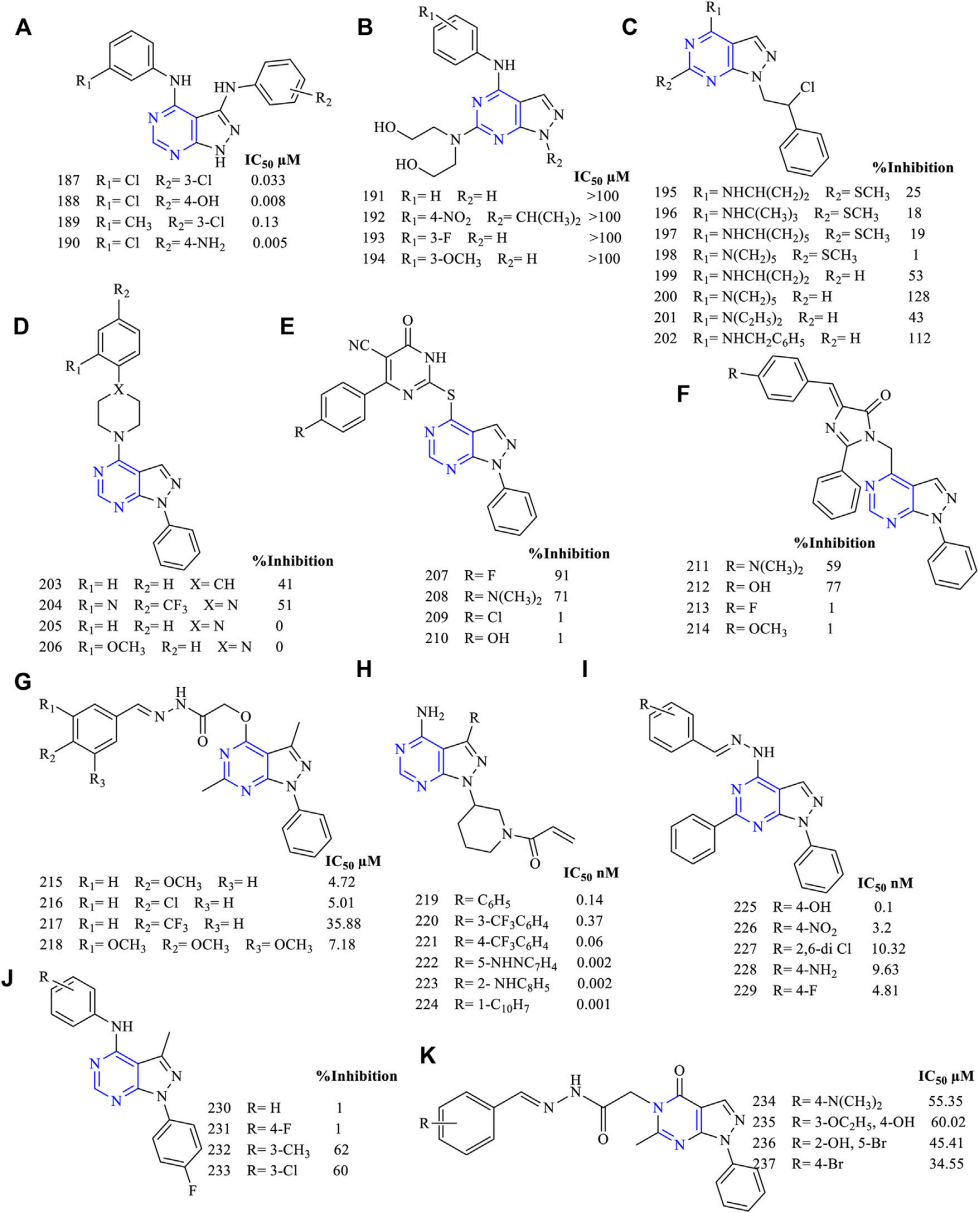
Chemical structure of **(A)** 4-(phenylamino) pyrazolo[3,4-*d*]pyrimidine pyrazolopyrimidine, **(B)** 4-substituted anilino-6-hydroxylaminopyrazolo[3,4-*d*]pyrimidine, **(C)** 1-(phenyl ethyl)-4-substituted amino-6-thiopyrazolo[3,4-*d*]pyrimidine, **(D)** piperidine-substituted pyrazolo[3,4-*d*]pyrimidine, **(E)** pyrimidinone-substituted pyrazolo[3,4-*d*]pyrimidine, **(F)** imidazole-substituted pyrazolo[3,4-*d*]pyrimidine, **(G)** acetohydride derivatives of pyrazolo[3,4-*d*]pyrimidine, **(H)** propanone derivatives of pyrazolo[3,4-*d*]pyrimidine, **(I)** benzylidene derivatives of pyrazolo[3,4-*d*]pyrimidine **(J)** 1-phenyl-3-methylpyrazolo[3,4-*d*]pyrimidin-4-amine, and **(K)** *N′*-substituted benzylidene-2-(6-methyl-4-oxo-1-phenyl-1*H*-pyrazolo[3,4-*d*]pyrimidin-5(4*H*)-yl)acetohydrazide.

Based on the pharmacophoric studies, 4-(phenylamino) pyrazolo [3,4-*d*]pyrimidine (**187–190**, Figure 15A) (EGFR IC_50_ 0.033 µM for **187**, 0.008 µM for **188**, 0.13 µM for **189,** and 0.005 µM for **190**) was designed and synthesized. These compounds were subjected to cellular study using MK cell lines and A-431 cell lines and found to be highly active. It was further reported that the compounds have a pronounced effect on the blockage of the EGFR signaling pathway. Furthermore, the *in vivo* studies revealed that the compounds were effective in the mice model when the animal was intraperitoneally administered with 12.5 mg/kg and 50 mg/kg (Traxler et al., 1997).

It was reported that 4-substituted anilino-6-hydroxylaminopyrazolo [3,4-*d*]pyrimidine derivatives (**191–194,** Figure 15B) were synthesized as anti-EGFR inhibitors. However, they lacked specific anti-EGFR properties (IC_50_ of synthesized compounds was more than 100 µM, whereas for the standard compound, it was found to be 0.0008 µM). Instead, they were found to inhibit CDK2, which plays an important role in the cell cycle (Kim et al., 2003).

Schenone et al. were the first in designing 6-substituted pyrazolo [3,4-*d*]pyrimidine as an antiproliferative agent. They designed and synthesized various 1-(phenyl ethyl)-4-substituted amino-6-thiopyrazolo [3,4-*d*]pyrimidine derivatives (**195–198**, Figure 15C) (percentage EGFR inhibition: 25% for **195**, 18% for **196**, 19% for **197,** and 0% for **198**). These compounds were less cytotoxic and ineffective compared to the reference molecule AG1478 (Schenone et al., 2004b). Further, they removed the substitution on the sixth position (**199–202**, Figure 15C) (53% for **199**, 128% for **198**, 43% for **199**, and 112% for **200**), and it was found that the compounds were effective in inhibiting EGFR. Compound **200** was the most potent and showed its activity against EGFR by blocking ERK and SRC that was required for its downstream signaling pathway (Schenone et al., 2004a).

The activity of these compounds also varied depending on the type of moiety that is substituted on the pyrazolo [3,4-*d*]pyrimidine. Abbas et al. synthesized pyrazolo [3,4-*d*]pyrimidine with three different attachments on the piperidine (**203–206**, Figure 15D) (percentage EGFR inhibition: 41% for **203**, 51% for **204**, 0% for **205–206**), pyrimidinone (**207–210**, Figure 15E) (percentage EGFR inhibition: 91% for **207**, 71% for **208**), and imidazole (**211–214**, Figure 15F) (percentage EGFR inhibition: 59% for **211**, 77% for **212**) moieties. On comparing their inhibitory potential, it was found that pyrimidinone was the most active as they gave a maximum inhibition of 91% when compared to gefitinib. The docking was carried out using protein PDB ID: 3W2O. The co-crystallized ligand was able to form a hydrogen bond with the MET793 residue, whereas the pyrimidinone 203–206 was binding not only to MET793 but also to the LYS745 residue. These resulted in stronger binding between the molecules and the protein, exhibiting higher EGFR inhibitory potential (Abbas et al., 2015).

The acetohydride derivatives of pyrazolo [3,4-*d*]pyrimidine were designed and synthesized (**215–218**, Figure 15G) (EGFR IC_50_ 4.72 µM for **215**, 5.01 µM for **216**, 35.88 µM for **217,** and 7.18 µM for **218**). These compounds were highly active against the MCF-7 cell lines with IC_50_ values in the range of 6.14–15.21 µM. These compounds were effective against breast cancer cell lines. Furthermore, the docking analysis predicted stronger binding interactions of compound **218** with the protein as compared to the reference erlotinib, suggesting that it can be further modified to improve its activity and enter into preclinical studies (Abdelgawad et al., 2016).

A structure-guided synthesis of propanone derivatives (**219–224**, Figure 15H) (EGFR IC_50_ 0.14 nM for **219**, 0.37 nM for **220**, 0.06 nM for **221**, 0.002 nM for **222**, 0.002 nM for **223,** and 0.001 nM for **224**) was carried out. The compounds have shown greater inhibition potential to EGFR^L858R^ and EGFR^L858R/T790M^. These compounds were further screened against the third-generation EGFR inhibitor gefitinib and were found to be superior, concluding that the compounds synthesized were selective third-generation EGFR inhibitors. Further, **223** was taken up for preclinical study using the mice model and results showed good activity by the intraperitoneal route compared to that by intravenous (Engel et al., 2017).

Making use of rational drug design tools and methods, benzylidene derivatives of pyrazolo [3,4-*d*]pyrimidine (**225–229**, Figure 15I) (EGFR IC_50_ 0.1 nM for **225**, 3.2 nM for **226**, 10.32 nM for **227**, 9.63 nM for **228**, and 4.81 nM for **229**) were designed, synthesized, and evaluated. The compounds were tested against the MCF-7, A-549, and mutant T790M. All the synthesized molecules were effective against most of the cell lines, whereas **229** was the most potent. Compound **229** showed good binding interaction in the docking studies but failed to exhibit selectivity. Moreover, the cell cycle analysis has shown that these compounds were capable of inhibiting the G_0_/G_1_ phase of the cell division (Gaber et al., 2018).

The design and synthesis of 1-phenyl-3-methylpyrazolo [3,4-*d*] pyrimidin-4-amine (**230–233**, Figure 15J) (A549 inhibition: 1% for **230–231**, 62% for **232,** and 60% for **233**) derivatives were carried out, and their inhibitory studies were revealed. The compounds showed a dual inhibitory action against EGFR as well as to ErbB2, which was further confirmed by performing caspase-3 analysis. Also, cell cycle analysis revealed that compound **233** showed the cell division arrest by the G_0_/G_1_ phase (Maher et al., 2019).

The design of carbohydrazide-based *N*′-substituted benzylidene-2-(6-methyl-4-oxo-1-phenyl-1*H*-pyrazolo [3,4-*d*]pyrimidin-5(4*H*)-yl)acetohydrazide (**234–237**, Figure 15K) (MCF IC_50_ 55.35 ± 7.711 µM for **234**, 60.02 ± 2.716 µM for **235**, 45.41 ± 5.376 µM for **236,** and 34.55 ± 2.381 µM for **237**; EGFR IC_50_ 0.186 µM for **237** and 0.03 µM for erlotinib) has been reported. The compounds have displayed EGFR inhibition using cell lines. These compounds were also subjected to docking analysis using PDB ID: 1M17. It was found that these compounds could inhibit the ATP binding site of the protein when compared to the binding interaction of the co-crystallized ligand. Flow-cytometry studies have found that **235** exhibited the highest apoptosis activity followed by **234, 236,** and **237** when compared to the control 0.5% DMSO vehicle. This represents the apoptotic activity of these compounds (Horchani et al., 2021).

#### 3.4.1 SAR of Pyrazolopyrimidine

Depending on the types of substitutions, pyrazolo [3,4-*d*]pyrimidine**,** the following is the compiled data of SAR:

1) Pyrazolo [3,4-*d*]pyrimidine showed good inhibitory activity against EGFR.
2) At N^3^: the presence of the carbohydrazide group results in decreased EGFR inhibitory activity but improves selectivity in binding to EGFR (Horchani et al., 2021).
3) At R_1_: the presence of a phenyl group showed good inhibitory properties (Abbas et al., 2015; Gaber et al., 2018). Replacing with the dialkylamino group exhibited reduced activity (Kim et al., 2003), whereas replacing with the thio group resulted in a significant loss of activity (Schenone et al., 2004b).
4) At R_2_: the linker can be either S or N; this resulted in compounds with good inhibitory activity (Abbas et al., 2015). The presence of the NH_2_ group makes the compound active toward the double mutant EGFR (Engel et al., 2017). The *N*-phenyl compounds were also active (Traxler et al., 1997; Kim et al., 2003; Maher et al., 2019). Replacing phenyl with piperidine or imidazole increased the potency (Abbas et al., 2015). The presence of acetohydride linkage results in the enhanced binding of the compounds to EGFR (Abdelgawad et al., 2016), whereas the hydrazine reduces the selectivity of the compound to EGFR (Gaber et al., 2018).
5) At R_3_: the presence of an aniline group is responsible for the potent activity toward EGFR (Engel et al., 2017)**.** Replacing the aniline with methyl exhibited a dual inhibitory activity to EGFR and ErbB2 (Maher et al., 2019).
6) At R_4_: the presence of a piperidine ring gives potent activity (Engel et al., 2017). Substituting with phenyl decreases the activity of the compound (Abbas et al., 2015; Abdelgawad et al., 2016; Engel et al., 2017; Gaber et al., 2018; Maher et al., 2019), whereas the phenyl ethyl group improves the inhibitory activity (Schenone et al., 2004b; 2004a).

**FIGURE 16:**
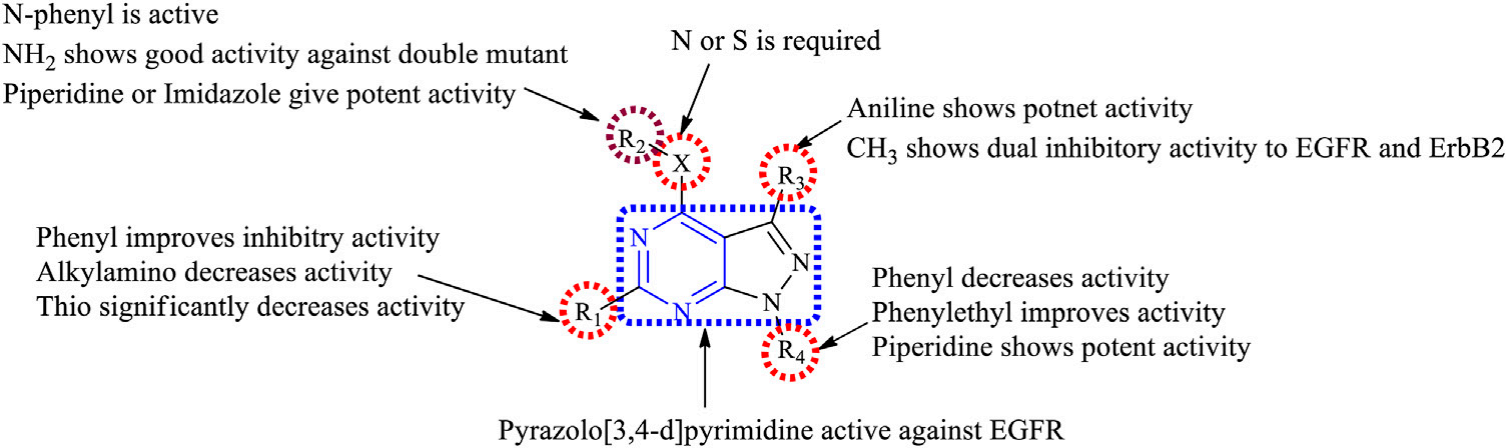
Summary SAR of pyrazolopyrimidine.

The summary of the SAR is depicted in Figure 16.

### 3.5 Furopyrimidines

Furan is a five-membered heterocycle that consists of one oxygen atom in the system. Substitution can occur in a total of four positions, depending on the fusion of furan and pyrimidine.

**FIGURE 17:**
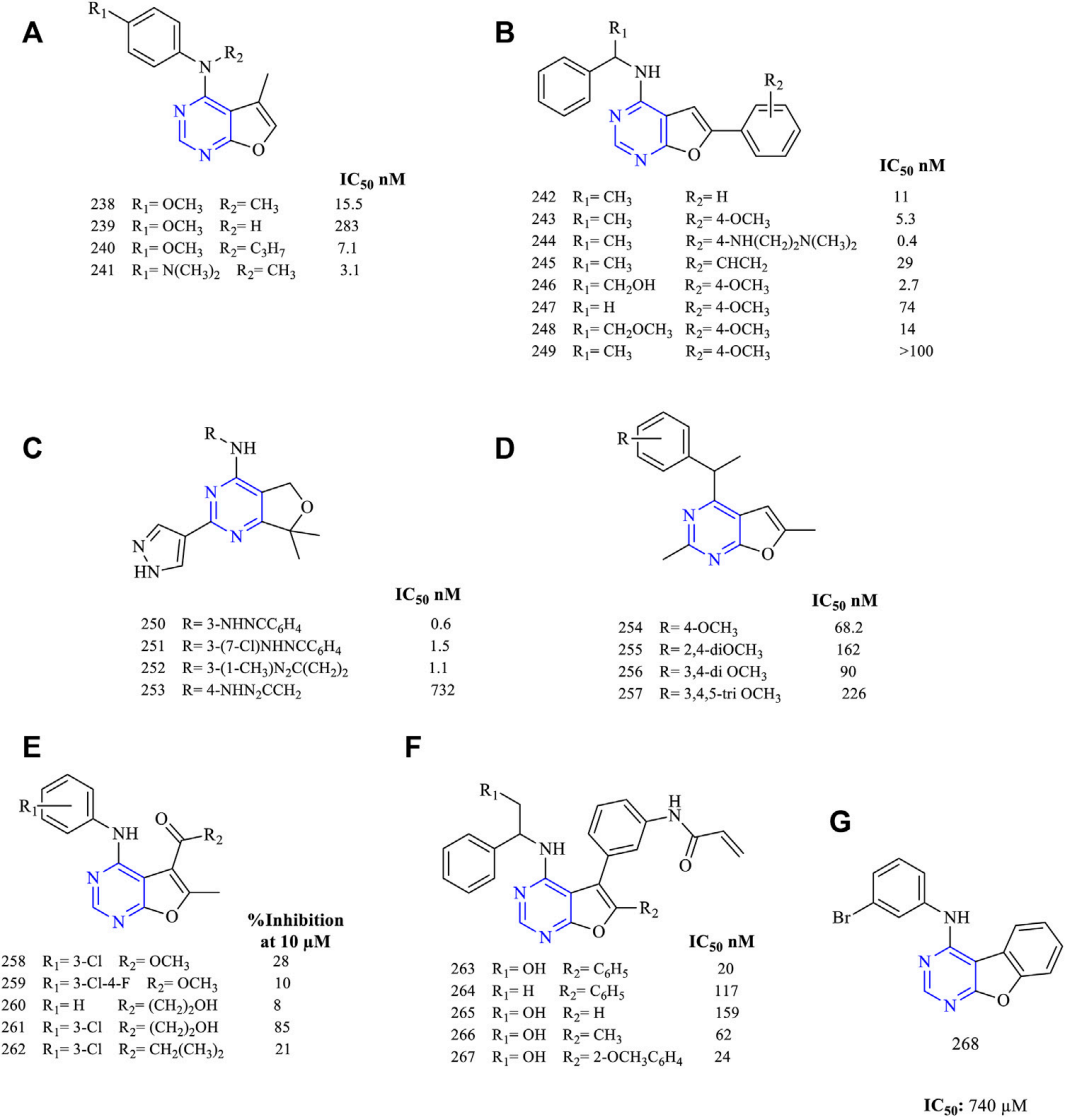
Chemical structure of **(A)** 4-amino-5-methylfuro[2,3-*d*]pyrimidine, **(B)** 6-arylsubstituted furo[2,3-*d*] pyrimidin-4-amine, **(C)** 2-indazolesubstituted furo[2,3-*d*] pyrimidin-4-amine, **(D)** 4-substituted-2,6-dimethylfuro[2,3-*d*]pyrimidine, **(E)** 4-anilino-6-methylfuro[2,3-*d*] pyrimidin-5-carboxylate, **(F)** propenamide derivatives of furo[2,3-*d*]pyrimidine, and **(G)** phenyl fused furo[3,2-*d*]pyrimidine.

It was reported that 4-amino-5-methylfuro [2,3-*d*]pyrimidine (**238–241**, Figure 17A) (EGFR IC_50_ 15.5 nM for **238**, 283 nM for **239**, 7.1 nM for **240,** and 3.1 nM for **241**) has been designed and studied for anti-EGFR activity. The compounds exhibited good kinase activity. The compounds were further tested for tubulin inhibitory activity, and **241** was found to be the most potent (Devambatla et al., 2018).

The 6-arylsubstituted furo [2,3-*d*] pyrimidin-4-amine (**242–249**, Figure 17B) (EGFR IC_50_ 11 nM for **242**, 5.3 nM for **243**, 0.4 nM for **244**, 29 nM for **245**, 2.7 nM for **246**, 74 nM for **247**, 14 nM for **248**)-based derivatives were synthesized with varied substitutions on the sixth position. The kinase inhibition studies found that the presence of ethyl amino linker in **243** at the sixth position is very effective and potent (IC_50_ 0.4 nM), whereas other substitutions decreased the activity. Compound **243** was subjected to further studies to identify its cellular potency but showed a significant loss in cellular activity (IC_50_ 217 nM) when compared to erlotinib (IC_50_ 87 nM) (Han et al., 2016b).

Based on the design and pharmacophoric groups, the indazole group has been tried (**250–253,** Figure 17C) (EGFR IC_50_ 0.6 nM for **250**, 1.5 nM for **251**, 1.1 nM for **252,** and 732 nM for **253**). These compounds with the triazole moiety exhibited great inhibitory potential toward EGFR with an IC_50_ in the range of 0.6–1.5 nM. Also, these compounds were found to be potent against the mutant cell line EGFR^L858R/T790M^ (Hanan et al., 2016).

Depending on the conformational studies, 4-substituted-2,6-dimethylfuro [2,3-*d*]pyrimidine (**254–257** Figure 17D) (EGFR IC_50_ 68.2 nM for **254**, 162 nM for **255**, 90 nM for **256,** and 226 nM for **257**) were synthesized and studied. These compounds were less effective against EGFR and VEGFR but were capable of inhibiting the tubulin network (Zhang X. et al., 2015).

A series of 4-anilino-6-methylfuro [2,3-*d*] pyrimidin-5-carboxylate (**258–262**, Figure 17E) (percent EGFR inhibition at 10 µM: 28% for **258**, 10% for **259**, 8% for **260**, 85% for **261,** and 21% for **262**)-based derivatives were synthesized. These compounds were capable of showing a weak dual inhibitory activity toward EGFR and ErbB2. Also, the antiproliferative studies proved that the compounds exhibited a significant loss of activity (Hossam et al., 2018).

The literature review suggests that the propenamide derivatives (**263–267**, Figure 17F) (EGFR IC_50_ 20 nM for **263**, 117 nM for **264**, 159 nM for **265**, 62 nM for **266,** and 24 nM for **267**) were designed and synthesized and these were highly effective against the EGFR^WT^ and the double mutant EFGR^L828R/T790M^. Moreover, these compounds have been tested with poziotinib (a potent inhibitor of HER2 exon 20 insertions under the clinical trial phase II (NCT03066206). It was found that the propenamide derivatives exhibited equal potency as compared to poziotinib in inhibiting EGFR and HER2 (Lin et al., 2019).

From the literature, it was found that the furo [3,2-*d*]pyrimidine can be fused with a phenyl ring (**268**, Figure 17G). This fusion led to a significant loss in the inhibitory activity toward EGFR with an IC_50_ of 740 µM (Showalter et al., 1999). These types of compounds were not showing much biological activity.

#### 3.5.1 SAR of Furopyrimidine

Various furopyrimidines have been studied. Based on the results reported in the literature, most of these compounds showed the presence of [2,3-*d*] fusion between furan and pyrimidine. This section provides an overview of the SAR:

1) The furo [2,3-d]pyrimidine derivatives were active against EGFR but modifying to furo [3,4-*d*]pyrimidine produced potent and selective EGFR inhibition (Hanan et al., 2016).
2) At R_1_: the presence of the CH_3_ group resulted in a decreased EGFR inhibitory activity (Zhang X. et al., 2015), whereas substituting R_1_ with the imidazolyl ring resulted in potent EGFR inhibitors that were active against the double mutant cell lines (Hanan et al., 2016).
3) At R_2_: the presence of a secondary N is required. Converting it into tertiary N, the activity was found to decrease (Zhang X. et al., 2015). Amino phenyl and methoxy-substituted phenyl derivatives were found to be active (Devambatla et al., 2018), but di- and tri-substituted methoxy phenyl compounds showed significant loss of activity (Zhang X. et al., 2015). A carbon linker present between the phenyl and N helps in improving its activity (Han et al., 2016b; Lin et al., 2019). Substituting with triazole shows a significant loss in anticancer activity, whereas indazole is a potent inhibitor (Hanan et al., 2016).
4) At R_3_: methyl substituents were found to be active (Devambatla et al., 2018). The carboxylate derivatives showed a significant loss of activity (Hossam et al., 2018). The presence of a phenyl ring decreases anticancer activity (Lin et al., 2019).
5) At R_4_: substitution with a phenyl ring showed good inhibitor activity (Han et al., 2016b). Fusing with benzene slightly reduces its activity (Showalter et al., 1999). Replacing with the methyl group significantly decreases the inhibitory activity (Hossam et al., 2018; Lin et al., 2019).

**FIGURE 18:**
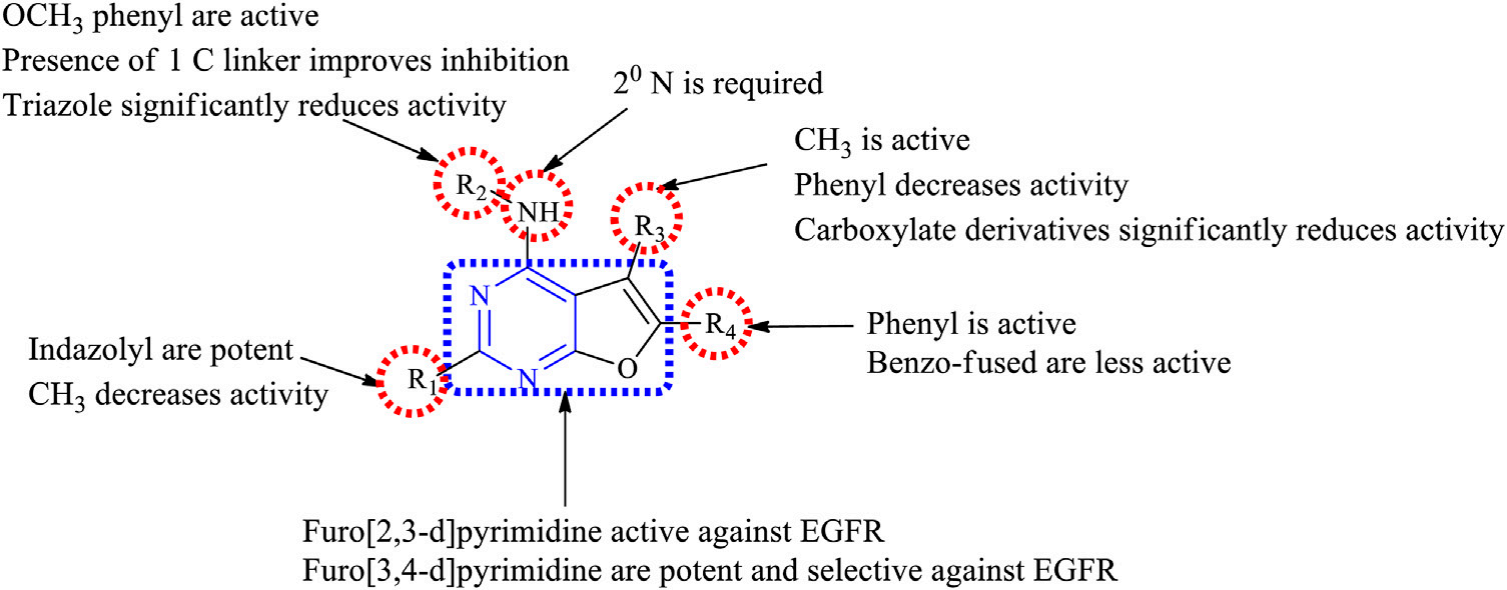
Summary SAR of furopyrimidine.

The summary of the SAR is depicted in Figure 18.

### 3.6 Pyrimidopyrimidines

Pyrimidine is a six-membered heterocycle that is fused with its counterpart pyrimidine with [4,5-*d*] fusion and [5,4-*d*] fusion. This fusion greatly influences the EGFR inhibitory activity of the compounds.

**FIGURE 19:**
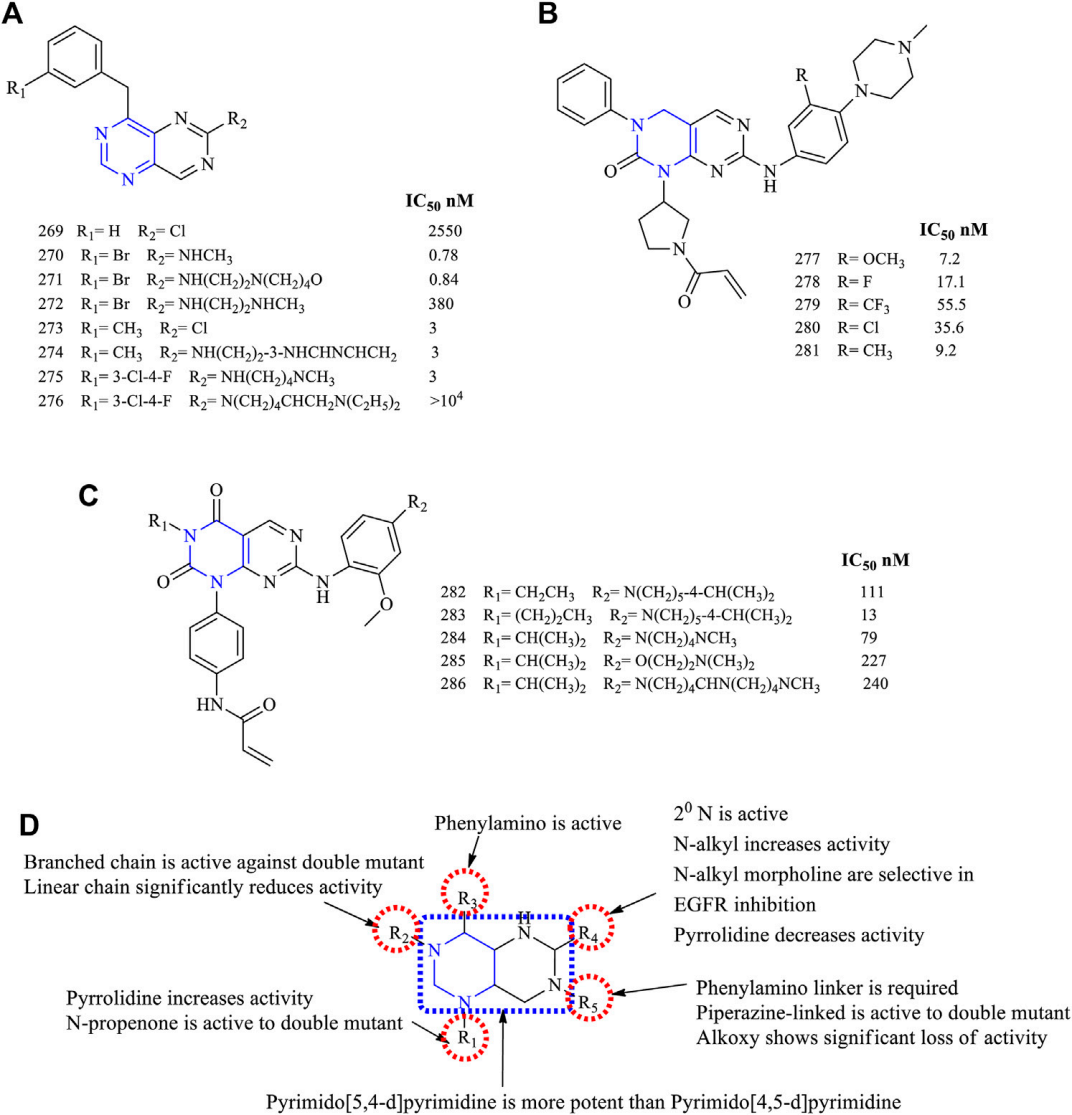
Chemical structure of **(A)** 4-amino-6-substituted pyrimido[5,4-*d*]pyrimidine, **(B)** 2-oxo-3,4-dihydropyrimido[4,5-*d*]pyrimidine, **(C)** pyrimido[4,5-*d*] pyrimidin-2,4-(1H,3H)-dione, and **(D)** summary SAR of pyrimido[5,4-*d*]pyrimidine.

The 4-amino-6-substituted pyrimido [5,4-*d*]pyrimidine **269–276**, Figure 19A) (EGFR IC_50_ 2,550 nM for **269**, 0.78 nM for **270**, 0.81 nM for **271**, 380 nM for **272**, 3 nM for **273**, 3 nM for **274–275**)-based derivatives were synthesized and reported to have an anti-EGFR activity. These compounds were tested against the A431 cancer cell line for their antiproliferative activity, and the results indicated that they were potent with IC_50_ values in the range of 0.78–3 nM (Rewcastle et al., 1997; Solca et al., 2004). The nitrogen at position 5 resulted in enhancing its activity by increasing the interaction with the target (Rewcastle et al., 1997). Compounds **274** and **275** were studied further for their antitumor activity using the mice xenograft model. Compound **274** was found to be more effective in reducing the tumor size, and Western blot analysis revealed that it was selective in inhibiting EGFR phosphorylation (Solca et al., 2004).

A series of 2-oxo-3,4-dihydropyrimido [4,5-*d*]pyrimidine (**277–281**, Figure 19B) (EGFR IC_50_ 7.2 nM for **277**, 17.1 nM for **278**, 55.5 nM for **279**, 35.6 nM for **280,** and 9.2 nM for **281**)-based derivatives were synthesized and its inhibitory properties were investigated. These compounds were found to inhibit EGFR in a range of IC_50_ 7.2–35.6 nM. Also, it was found that the compound with a bulkier substituent CF_3_ was less potent than the others. Further, compound **280** was found to be better in terms of the pharmacokinetic properties and antitumor activities in the Sprague-Dawley rat model, whereas the Western blot analysis proved that **280** was an irreversible inhibitor of EGFR and resulted in the complete blockage of the EGFR pathway (Xu et al., 2013).

Based on the molecular hopping, mutant selective pyrimido [4,5-*d*] pyrimidin-2,4-(1H, 3H)-dione was synthesized (**282–286**, Figure 19C) (EGFR IC_50_ 111 nM for **282**, 13 nM for **283**, 79 nM for **284**, 227 nM for **285,** and 240 nM for **286**). The compounds were investigated against EGFR^WT^ and EGFR^L858R/T790M^, and the results indicated that they were more selective to the mutant form. When compared using the reference AZD9291, the time of onset and duration of action were similar to **284**, but in the A431 xenograft model, **286** showed less inhibition in tumor growth (Hao et al., 2018).

#### 3.6.1 SAR of Pyrimidopyrimidines

Based on the available literature, pyrimidopyrimidines can undergo two types of fusion, mainly pyrimido [4,5-*d*] pyrimidine and pyrimido [5,4-*d*]pyrimidine. The following section gives the compilation of the SAR:

1) The fused pyrimido [5,4-*d*]pyrimidine was found to be more potent than the pyrimido [4,5-*d*]pyrimidine fused.
2) At R_1_: the presence of pyrrolidine helps in improving the solubility and pharmacokinetics of the compounds (Xu et al., 2013). Substitution with *N*-propenone makes the compound selective against double mutant EGFR (Hao et al., 2018).
3) At R_2_: the short alkyl chain of two to three carbons was investigated. The linear chains showed a significant loss in inhibitory activity, whereas the branched-chain (Hao et al., 2018) and phenyl substituents (Xu et al., 2013) were found to be potent against the double mutant.
4) At R_3_: the phenylamino is required for its anti-EGFR activity (Rewcastle et al., 1997; Solca et al., 2004).
5) At R_4_: chlorine decreases the activity (Rewcastle et al., 1997). The secondary N is required for the activity (Rewcastle et al., 1997; Solca et al., 2004). When *N* is a part of the heterocycle system, the activity decreases (Solca et al., 2004). The *N*-alkyl substituents were active. Also, *N*-alkylmorpholine substituents were selective in inhibiting EGFR, whereas alkyl diamino was less active (Rewcastle et al., 1997).
6) At R_5_: the phenylamino was linked with heterocycle piperidine, and the inhibitory activity decreased (Hao et al., 2018). Replacing piperidine with piperazine, the activity of compounds increased and they are more selective against the double mutant EGFR (Xu et al., 2013; Hao et al., 2018), whereas replacing with alkoxy resulted in significant loss of activity (Hao et al., 2018).

The summary of SAR is depicted in Figure 19D.

### 3.7 Pyrimidoindole

Indole is a heterocycle that consists of benzene fused with pyrrole. Based on the literature, pyrimidoindole was synthesized; also, the available literature suggests that pyrimidoindole is less active against EGFR.

**FIGURE 20:**
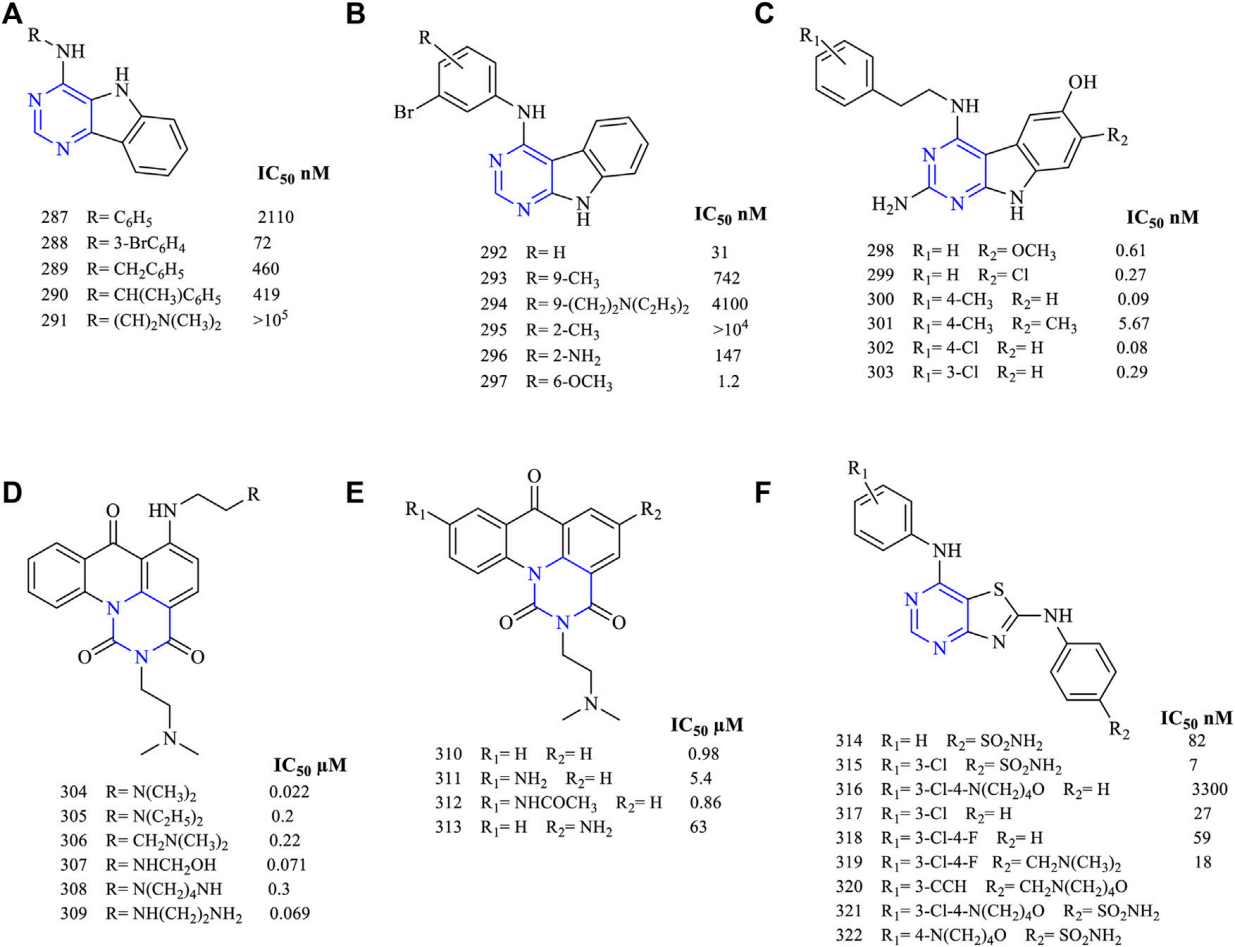
Chemical structures of **(A)** pyrimido [5,4-*b*]indol-4-amine, **(B)** *N*-phenylpyrimido [5,4-*b*]indol-4-amine, **(C)** 2-amino-4-(phenylethylamino) pyrimido [4,5-*b*]indole, **(D)** 6-substituted pyrimido [5,6,1-de]acridin-1,3,7-trione, **(E)** 9-substituted pyrimido [5,6,1-*de*]acridin-1,3,7-trione), and **(F)** 4,6-diaminothiazolo [4,5-*d*]pyrimidine.

A series of pyrimido [5,4-*b*] indol-4-amine (**287–291**, Figure 20A) (EGFR IC_50_ 2,110 nM for **287**, 72 nM for **288**, 460 nM for **289**, 419 nM for **290,** and >10^5^ nM for **291**) derivatives were synthesized. These compounds were tested against the A431 cell lines, and studies showed that these molecules had inhibitory potential. These compounds further exhibited a significant loss of activity toward EGFR because of the lack of interaction with the target cell (Showalter et al., 1999). Similarly, *N*-phenylpyrimido [5,4-*b*] indol-4-amine (**292–297**, Figure 20B) (EGFR IC_50_ 31 nM for **292**, 742 nM for **293**, 4,100 nM for **294**, >10^4^ nM for **295**, 147 nM for **296,** and 1.2 nM for **297**)-based derivatives were also synthesized. Among them, compound **297** was the only potent molecule found (IC_50_ 1.2 nM), whereas others lacked activity toward EGFR. This loss of activity was due to the presence of an electron-withdrawing group present in the ring (Showalter et al., 1999).

The compound 2-amino-4-(phenylethylamino) pyrimido [4,5-*b*] indole (**298–303**, Figure 20C) (EGFR IC_50_ 0.61 nM for **298**, 0.27 nM for **299**, 0.09 nM for **300**, 5.67 nM for **301**, 0.08 nM for **302,** and 0.29 nM for **303**) has been designed and studied. These compounds were found to inhibit EGFR in the nanomolar range, making them very potent and selective in inhibition. Further, compounds **300** and **302** were found to be active against VEGFR2. The growth inhibition of **298** against MDA-MB-231 cell lines is 34%, whereas that for **300** is 65%. These compounds were highly active due to the presence of the phenylamino substituent in the fourth position (Fischer et al., 2017)**.**

### 3.8 Pyrimido Acridine

Acridine was fused with pyrimidines, and the inhibitory potential was determined against the EGFR receptor. In order to inhibit DNA replication, 6-substituted pyrimido [5,6,1-*de*] acridin-1,3,7-trione (**304–309**, Figure 20D) (HT29 IC_50_,0.022 µM for **304**, 0.2 µM for **305**, 0.22 µM for **306**, 0.071 µM for **307**, 0.3 µM for **308,** and 0.069 µM for **309**) has been designed. They have been tested for their antitumor activity against HT29 and LoVo/DX resistant cell lines. These compounds were found to be effective in inhibiting both the cell lines, whereas **302** was the most potent against HT29 (IC_50_ 0.022 µM) and LoVo/DX (IC_50_ 0.029 µM). Further, the cytotoxicity studies revealed that the basic side chain was essential for its antitumor activity. Also, the presence of hydrogen bonding between the 4-N and 5-O improved the cytotoxicity profile and DNA binding (Antonini, 2002).

Further, it was reported that the 9-substituted pyrimido [5,6,1-*de*] acridin-1,3,7-trione (**310–313** Figure 20E) (P388 IC_50_ 0.98 µM for **310**, 5.4 µM for **311,** 0.86 µM for **312**, and 63 µM for **313**) derivatives were tested for their cytotoxicity against various cancer cell lines. Based on the results, the substitution at the ninth position exhibited potent to weak activity. Further *in vivo* studies were carried out using P388 leukemia in mice model, and **310** was found to be capable of increasing the life expectancy at a dose of 25 mg/kg/day but the animals died due to toxicity at such a high dose (Kamata et al., 2004). But the acridine-based derivatives were not recommended anymore to their high toxic nature.

### 3.9 Thiazolopyrimidines

Thiazole is a five-membered heterocycle consisting of heteroatoms sulfur and nitrogen. Based on the literature, thiazole is fused with pyrimidine by [4,5-*d*] fusion. Using molecular dynamics and molecular modeling studies, 4,6-diaminothiazolo [4,5-*d*]pyrimidine (**314–322**, Figure 20F) (IC_50_ 82 nM for **314**, 7 nM for **315**, 3,300 nM for **316**, 27 nM for **317**, 59 nM for **318**, 15 nM for **319**, 21 nM for **320**) (Dong et al., 2011; Mitrasinovic, 2014) were designed and studied for their anti-EGFR inhibition. Molecules **314–318** have been studied using docking analysis and were found to inhibit the ATP-binding site of EGFR. The SAR study further revealed that the 3′ and 4’ positions on R_2_ were beneficial in binding and the presence of chlorine enhanced its selectivity (Dong et al., 2011). Molecules **321** and **322** were structurally similar except with the chlorine substitution, but results indicated a difference in the activity of both the compounds. Also, it was found that **321** was a non-specific intercalator, whereas **322** was an intercalator and grove inhibitor as compared to lapatinib (Mitrasinovic, 2014).

### 3.10 Miscellaneous Pyrimidine-Based EGFR Inhibitor

This section provides details of the pyrimidine-based EGFR inhibitors that are not fused with a heterocyclic system. The literature suggests that the NH group on the pyrimidine ring is an important substituent for exhibiting EGFR inhibitory activity.

**FIGURE 21:**
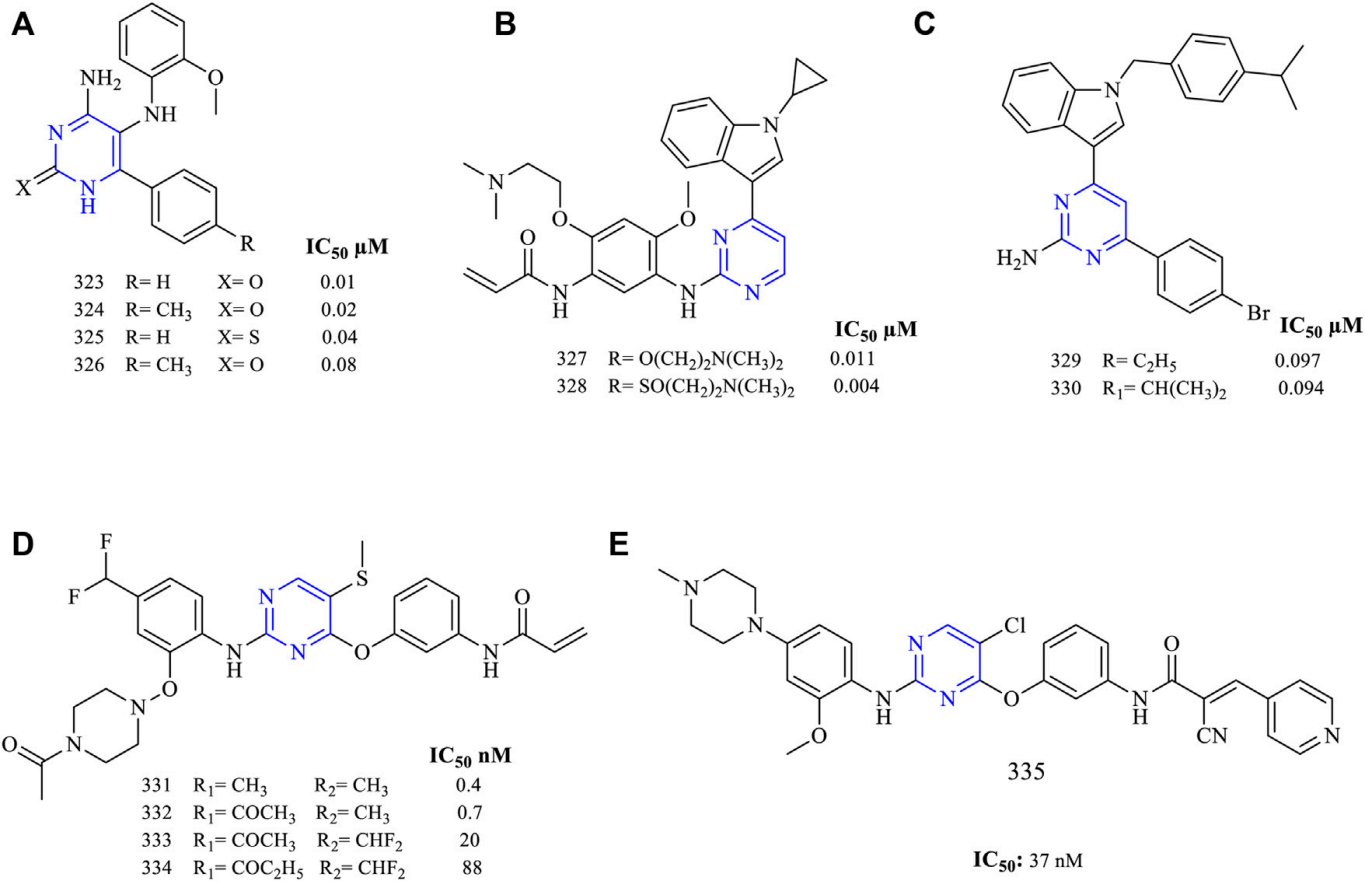
Chemical structures of **(A)** 4-amino-5-((2-methoxyphenyl)amino)-6-(4-substituted) phenylpyrimidin-2(1H)-one, **(B)** *N*-(5-((4-(1-cyclopropyl-1H-indol-3-yl)pyrimidin-2-yl)amino)-2-(substituted)-4-methoxyphenyl)acrylamide, **(C)** 4-(4-bromophenyl)-6-(1-(substituted)-1H-indol-3-yl)pyrimidin-2-amine, **(D)** *N*-(3-((2-((4-substituted methyl-2-((4-substituted piperazin-1-yl)oxy)phenyl)amino)-5-(methylthio)pyrimidin-4-yl)oxy)phenyl)acrylamide, and **(E)** *N*-(3-((5-chloro-2-((2-methoxy-4-(4-methylpiperazin-1-yl)phenyl)amino)pyrimidin-4-yl)oxy)phenyl)-2-cyano-3-(pyridin-4-yl)acrylamide.

A series of 4-amino-5-((2-methoxyphenyl) amino)-6-(4-substituted) phenylpyrimidin-2(1H)-one (**323–326,** Figure 21A) have been synthesized and studied for the EGFR inhibitory activity (EGFR^WT^ IC_50_: 0.087 ± 0.013 µM for **323**, 0.11 ± 0.014 µM for **324**). It was reported that these molecules were cytotoxic when analyzed using the MCF-7 cell line (0.01 ± 0.003 µM for **323**, 0.02 ± 0.001 µM for **324**, 0.04 ± 0.04 µM for **325**, 0.08 ± 0.02 µM for **326,** and 0.42 ± 0.2 µM for erlotinib, which was used as standard). The EGFR kinase inhibitory activity revealed that the molecules have displayed very little activity in inhibiting the EGFR^WT^ (Othman et al., 2021).

Another series of *N*-(5-((4-(1-cyclopropyl-1H-indol-3-yl)pyrimidin-2-yl)amino)-2-(substituted)-4-methoxyphenyl)acrylamide (**327–328**, Figure 21B) have been synthesized and their EGFR inhibitory potential was determined (H1975 IC_50_: 0.011 ± 0.001 µM for **327**, 0.004 ± 0.002 µM for **328,** and 0.01 ± 0.003 µM for erlotinib). The presence of the S atom in **328** has resulted in displaying a selectivity of 15.8 times toward EGFR^WT^ as compared to the erlotinib with a selectivity of 6.5. This inhibitory activity was a result of the presence of an indole ring in the molecule (Li et al., 2021).

A novel series of substituted indole were designed, as shown in Figure 21C (**329–330**). Their IC_50_ values were determined by using EGFR^WT^ (0.097 µM for **329**, 0.094 µM for **330,** and 0.9 µM for erlotinib). It was found that the molecules with two to three carbons in the substituent side chain of indole have exhibited higher EGFR inhibitory activity. Moreover, their EGFR kinase activity was found to be similar to that of erlotinib (Singh and Silakari, 2018) and hence such molecules are better antiproliferative agents rather than potent EGFR inhibitors.

A series of trisubstituted pyrimidine have been designed (Figures 21D,E). Both series differ in the position of the substituent, whereas the parent structure remains constant (EGFR^L858R/T790M^ IC_50_ values: 0.4 nM for **331**, 0.7 nM for **332**, 20 nM for **333**, 88 nM for **334,** and 37 nM for **335**). The higher EGFR inhibitory activity found for compounds **331** and **332** was due to the presence of “S” in the structure, whereas replacing “S” to “Cl” results in a drastic decrease in the EGFR inhibitory activity. This is due to the formation of stronger hydrophobic interactions with the MET790 residue present at the binding site of the protein. Moreover, the presence of the Michael acceptor helps improve the activity of the pyrimidine derivatives containing a heterocycle. These attributes have resulted in molecules that are selective in the inhibition of EGFR, as determined by using the Western blot analysis (Basu et al., 2015; Xiao et al., 2016).

### 3.11 Computational Aspects of Fused Pyrimidine as EGFR Inhibitor

**FIGURE 22:**
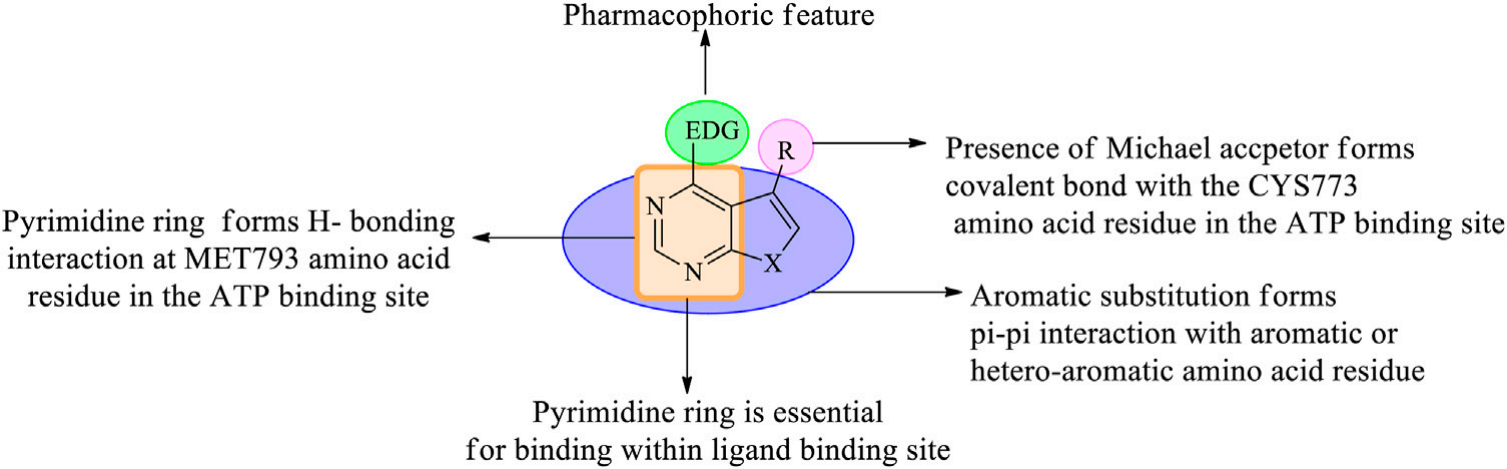
Computational aspects of fused pyrimidine as EGFR inhibitor.

Pyrimidine is essential for binding interaction with the ligand-binding site, as the ring fits into the ATP binding site forming H-bond with the MET793 residue (Aboulwafa et al., 2020). The fused-pyrimidine system consisting of an aromatic substitution results in the π–π interaction between the phenyl ring and the aromatic or hetero-aromatic amino acid residue. The presence of an electron-donating group on the pyrimidine ring is an important pharmacophore feature of the fused-pyrimidine to exhibit EGFR inhibitory activity. Moreover, the presence of the Michael acceptor results in the formation of a covalent bond to CYS773 present in the ATP binding site (Li and Li, 2014). See Figure 22.

### 3.12 Mechanism of Pyrimidine-Based Hybrids in Inhibiting EGFR

**FIGURE 23:**
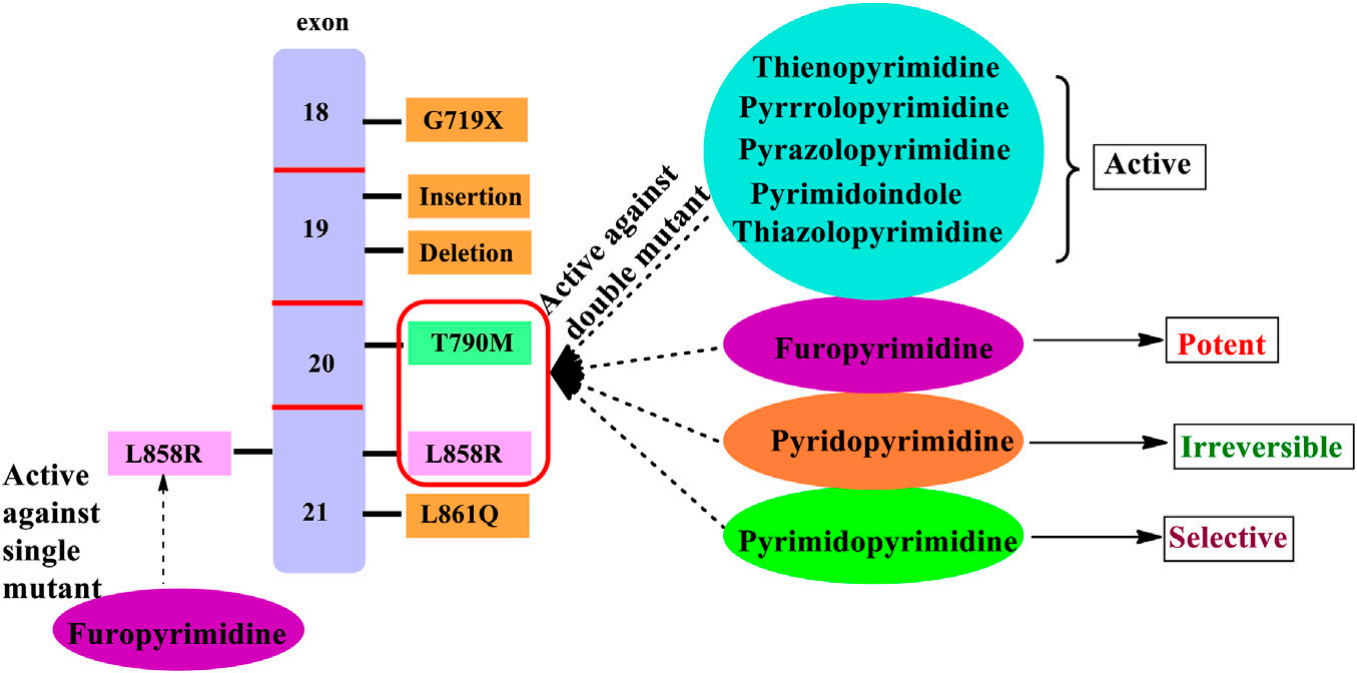
Mechanism of pyrimidine-based hybrids in inhibiting EGFR. The mutation mainly occurs at the T790M and L858R exons. From the known chemistry, various hybrids act differently on this mutated exon. Furopyrimidine shows inhibitory activity not only to L858R but also to the double mutant T790M and L858R. Pyridopyrimidine, thienopyrimidine, pyrrolopyrimidine, pyrimidopyrimidine, pyrimidoacridone, pyrazolopyrimidine, pyrimidoindole, and thiazolopyrimidine are active against the double mutant T790M and L858R. Pyridopyrimidine is the irreversible inhibitor, whereas pyrimidopyrimidine is a selective inhibitor.

From the *in vitro* studies, it is obvious that the pyrimidine derivatives display good inhibitory potential in inhibiting EGFR’s overexpression. The pyrimidine-fused hybrids mainly act on the mutation that takes place at L858R and T790M as mentioned above in this review, but their activities vary greatly with the structure and various changes in the heterocyclic ring and their respective substitution (see Figure 23). EGFR’s overexpression is a result of a mutation in the exon at the 20th position (T790M) and 21st position (L858R). The pyrimidine hybrids act on these two positions with different potencies. Furan shows inhibitory activity not only to single mutant L858R but also to the double mutant T790M and L858R. Pyridine, thiophene, pyrrole, pyrimidine, acridone, pyrazole, indole, and thiazole are active against the double mutant T790M and L858R. Pyridine is the irreversible inhibitor, whereas pyrimidine is a selective inhibitor.

## 4 Conclusion

EGFR is an important receptor that controls the cell growth and its proliferation. The uncontrolled downstream signaling of EGFR is the main cause underlying for uncontrolled cell growth leading to NSCLC. Various EGFR inhibitors like lazertinib, avitinib, nazartinib, osimertinib, erlotinib, gefitinib, afatinib, and lapatinib have been approved. They reduce the downstream signaling by inhibiting the PTK, and hence, there’s reduction in the cell proliferation. Based on the pharmacophoric features and SAR analysis, various researchers have tried exploring various chemical moieties for inhibiting EGFR. The most widely studied chemical structure is a pyrimidine. This review mainly focuses on the pyrimidine-fused heterocycle system. Pyridine, pyrrole, thiophene, pyrazole, furan, indole, acridone, thiazole, and pyrimidine are among these heterocycles. Certain heterocycles (pyridine, pyrrole, furan, and pyrazole) show stronger effects toward EGFR inhibition, while pyrrole and pyrazole have good potency against ErbB2, making them dual inhibitors, based on their inhibitory values and kinase studies. However, it is clear that thiophene, indole, and thiazole have a lower inhibitory potential for EGFR. In comparison to the merely substituted pyrimidine, the EGFR inhibitory efficacy of pyrimidine-based heterocyclic hybrids differs dramatically. It is also reported that pyrimidine hybrids shows inhibition toward EGFR and CDK. Therefore, the selectivity aspects come into picture. Selectivity in inhibiting EGFR is due to the presence of O in furan, S in thieophene, N in thiazole and indole, acridone (tricyclic ring system). Pyrimidine fused with such monocyclic heterocycle system and containing N, namely, pyrazole, pyrrole, pyrimidine, and pyridine, shows a dual inhibitory activity toward EGFR and CDK. Furthermore, because pyrimidine-based heterocyclic hybrid inhibitors have been proven to be very hazardous in *in vivo* investigations, it is critical to understand their toxicity profiles. SAR is an important tool in designing the molecules. Based on the review, it is quite evident that the studies should aim at bringing newer and better molecules using the available SAR of such pyrimidine analogs. Moreover, they should be thoroughly investigated for acute and chronic toxicity before being considered as potential EGFR inhibitors in the future. Using SAR of compounds will aid the researchers in the better designing of the medicinal molecules and greater insights into the development of powerful molecules with higher activity and fewer side effects, hence increasing the quality of life of patients with cancer.
